# Large-Scale Meta-Analysis of Nanomaterials Toxicity Based on Natural Language Processing of Scientific Articles

**DOI:** 10.1021/acsanm.5c05119

**Published:** 2025-12-23

**Authors:** Amauri J. Paula, Romana Petry, James M. Almeida, André A. Caetano, José Sales, Odair P. Ferreira, Diego S. T. Martinez, Henry J. Kobs, Andreia F. Faria

**Affiliations:** † Solid-Biological Interface Group (SolBIN), 28121 Aeronautics Institute of Technology - ITA, São José dos Campos 12228-900, SP, Brazil; ‡ Brazilian Nanotechnology National Laboratory (LNNano), 215006 Brazilian Center for Research in Energy and Materials (CNPEM), Campinas 13083-100, SP, Brazil; § Ilum School of Science, Brazilian Center for Research in Energy and Materials (CNPEM), Campinas 13087-548, SP, Brazil; ∥ Laboratory of Advanced Functional Materials (LaMFA), Department of Chemistry, State University of Londrina (UEL), Londrina 86055-900, SP, Brazil; ⊥ Engineering School of Sustainable Infrastructure & Environment, Department of Environmental Engineering Sciences, 3463 University of Florida, Gainesville 32611-6580, Florida, United States

**Keywords:** nanotoxicology, natural language processing, large language models, Ag, ZnO, nanoparticles, Escherichia coli, Staphylococcus aureus

## Abstract

Natural language processing (NLP) pipelines can mine the nanotoxicology literature at a scale and resolution that cannot be achieved by manual curation. Here, we established a NLP pipeline that coupled topic modeling and end point-specific extraction LLM prompts to convert ∼10^6^ sentences extracted from abstracts of scientific articles into a structured knowledge base containing 13 nanotoxicology features. The pipeline is capable of analyzing and extracting nanomaterial descriptors such as size, ζ potential, and surface area, along with biological end points such as minimum inhibitory concentration (MIC), minimum bactericidal concentration (MBC) and lethal concentration 50% (LC_50_). Statistical convergence across multiple quantitative end points - MIC, MBC, microbial log reduction, and biofilm killing efficiency - shows that Ag-based nanomaterials are the most potent antimicrobial agents, showing lower MIC and MBC than ZnO, TiO_2_ and Au analogs. This trend was also observed for individual pathogens such as *Escherichia coli* and *Staphylococcus aureus*. Most nanomaterials are within 1 to 100 nm, with nanoparticles featured in >80% of the studies. Although nanomaterials <50 nm often produce the lowest MIC and LC_50_, toxicity within a single size class spans orders of magnitude, underscoring the influence of surface chemistry, coatings, and colloidal behavior. In addition, adverse reproductive effects in *Caenorhabditis elegans* and *Daphnia magna*, and developmental abnormalities in *Danio rerio*, are predominantly related to Ag and TiO_2_. In general, our automated data extraction and the curation strategy transforms disparate literature into a machine-readable knowledge base that paves the way for data-driven predictions of nanomaterial hazards.

## Graphical abstract

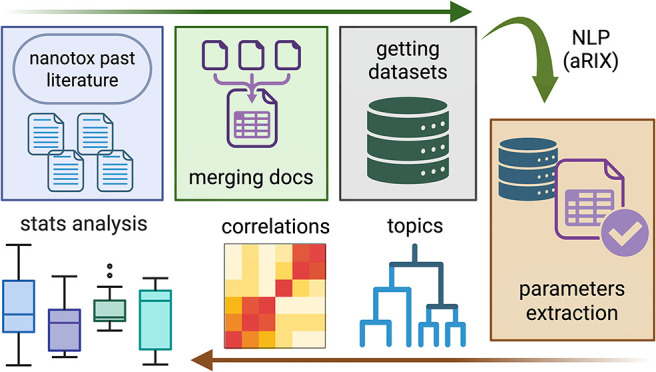

## Introduction

Advancements in nanotechnology have led to numerous scientific breakthroughs in our society, especially over the last 25 years, affecting areas from electronics to medicine.^1–3^ In a general sense, nanotechnology deals with the control of matter and its interactions at the nanoscale. More precisely, nanotechnology involves applying scientific expertise to harness the unique properties of materials at the nanoscale (up to 100 nm), which differ significantly from those of individual atoms, molecules, or the same material in larger dimensions (bulk scale).^4^ Nanomaterials are the primary products of nanotechnology. They are defined as materials having any external dimension in the nanoscale or having an internal structure or surface structure in the nanoscale. Nanomaterials include nano-objects and nanostructured materials.^5^ Nano-objects can be classified as (i) nanoparticles (NPs), which have three outer dimensions on the nanoscale; (ii) nanofibers, with two outer dimensions on the nanoscale; and (iii) nanoplates, with one external dimension in the nanoscale.^6^ Examples of inorganic nanoparticles include metal nanoparticles (silver, gold, and iron) and quantum dots, while organic nanoparticles include liposomes, micelles, and polymer-based ones. In addition, the class of nanofibers includes carbon nanotubes and inorganic nanotubes, such as titanium and vanadium oxide. Finally, nanoplates include graphene and other 2D materials.^7^ On the other hand, nanostructured materials are defined as materials that have an internal or surface nanostructure and include nanostructured powders, nanocomposites, and nanoporous materials.^8^

Nanomaterials can originate from three primary sources: (i) incidental, as byproducts of human-made processes; (ii) engineered, intentionally manufactured for specific tasks; and (iii) naturally produced, formed through natural environmental processes or within living organisms without human intervention.^9^ The deliberate release of engineered nanomaterials includes the use of iron oxide nanoparticles (Fe_*x*_O_*y*_, with Fe­(II) and Fe­(III)) to remediate contaminated soils and groundwater;^10^ titanium dioxide (TiO_2_) nanoparticles in sunscreens added;^11^ silica nanoparticles (SiO_2_) in fabrics and cosmetics;^12,13^ and polymeric/liposome nanoparticles for cancer treatment.^14,15^ In addition, nanomaterials based on zinc oxide (ZnO), copper oxide (CuO), cerium dioxide (CeO2), carbon nanotubes (CNTs), graphene, and quantum dots (CdSe, CdTe, PbSe) were intensively studied.^16–18^ However, the highest number of nanotechnological products so far has involved the use of silver nanoparticles (AgNPs), which are known for their strong antimicrobial activity.^19–21^ These products range from wound dressings, socks and other textiles, air filters, toothpaste, baby products, vacuum cleaners, and air conditioning devices and washing machines.^22–26^

The rapid advancement of engineered nanomaterials has raised concerns about their environmental and health impacts, which, in turn, have influenced their conversion to commercial products.^27^ In response, the field of nanotoxicology has emerged to investigate the potential risks associated with nanomaterials, highlighting the need for systematic studies to identify adverse effects and ensure safety.^28,29^ Great research efforts have been made to connect the main physicochemical properties of nanomaterials not only to their functionalities but also to their environmental fate, considering exposure to humans and other organisms. It is important to consider the toxic effects of these materials to assess their impact on living organisms in both terrestrial and aquatic environments.^9^ Nanomaterials can disrupt organism development by interfering with normal physiological functions, potentially causing malformations that can be fatal to embryos and growing animals.^30^ Physicochemical properties such as size and zeta potential play a critical role in chemical, physical, and biological interactions with living systems.^31^ It is known that smaller nanoparticles can easily penetrate cell membranes.^32^ This can alter cell metabolism and ultimately lead to cell death.^33^ In general, the toxicity of nanomaterials, particularly nano-objects, depends on multiple factors such as dose, exposure time, concentration, and the characteristics of the medium in which they are dispersed. In addition, size and shape-dependent toxicity has been documented in numerous studies, while surface area also plays a significant role in determining toxicity.^34^ These findings underscore that the evaluation of the impact of nanomaterials on biological systems requires the consideration of a wide range of variables. This is the reason why understanding and predicting the nanotoxicity is extremely challenging.

There is a vast scientific literature on toxicological assays in which tens of nanomaterials were tested against biological species present in soil, air, and water matrices. These assays have standardized end points with measurable biological, biochemical, or physiological effects, and can offer important insights into how nanomaterials interact with biological systems at multiple levels (e.g., molecular, cellular, organism, and ecological).^22,35–37^ In addition, the presence of tens of thousands of publications in the field presents both an opportunity and a challenge for researchers aiming to extract meaningful insights. Natural Language Processing (NLP) techniques offer a powerful solution for analyzing this extensive corpus. By automating the extraction of key information in the field, we can identify trends and uncover patterns within the literature.^38,39^ For example, NLP tools can classify studies, extract data on the properties of nanomaterials, and even suggest materials for certain applications.^40^ These techniques enable meta-analysis and topic modeling, which collectively contribute to a comprehensive overview of current research directions, knowledge gaps, and emerging topics, and can support decision making. Taking into account recent advances in materials science, NLP approaches have enabled new data-oriented processes, protocols, and predictive models to improve the area of battery materials,^41,42^ choose solvents for crystallization of molecules,^43^ structure polymer-property databases,^44^ provide rational reviews and prospects in pharmaceutical science,^45^ orient biochar synthesis,^46^ and provide information on catalysts acting in CO_2_-reduction.^47^

In this article, we have processed more than 50,000 abstracts of scientific articles related to nanotoxicology with an NLP approach. More specifically, we sought documents that describe toxicity assays using common biological models such as *Arabidopsis thaliana*, *Bacillus subtilis*, *Caenorhabditis elegans*, *Danio rerio*, *Daphnia magna*, *Drosophila melanogaster*, *Escherichia coli*, Green algae, rainbow trout, *Saccharomyces cerevisiae*, *Staphylococcus aureus*, and more than ten other species. The most common end points related to nanomaterial characteristics, such as composition, shape, size, zeta potential, and surface area, were correlated with toxicological assay end points such as antimicrobial activity, mortality and survival rates, and others. We used a previously described NLP pipeline^46^ in addition to large language models (LLMs) to perform an efficient extraction of relevant end points. LLMs have been shown to have an unmatched capacity to extract both (i) numerical parameters, e.g., nanomaterial size, zeta potential, and percentage of microbe reduction; and (ii) categorical parameters, e.g., nanomaterial composition and shape, and biological model species.^44,48^ The results allowed for a broad statistical analysis of the field and important comparisons about the possible environmental impacts of common nanomaterials, such as silver-, gold-, copper-, titanium- and zirconium-based, graphene, quantum dots, and 2D materials, across multiple biological levels.

## Experimental Section

### Data Acquisition and Text Processing

We used the Clarivate Web of Science (WOS) database - accessible through the Brazilian Periódicos CAPES portal - to locate 53,335 primary research articles (published up to 2024) on nanotoxicity with a carefully curated keyword set (see Supporting Information). For each WOS record, we captured the article’s Digital Object Identifier (DOI) and then query the Crossref platform, which returned the authoritative abstract together with complete bibliographic metadata (title, authors, affiliations, publication date, journal, volume, issue, and keywords) in tabular form. This search pattern was more efficient than using general terms such as “*toxic*”, which would find terms such as toxicity, toxic, nanotoxicity or nanotoxic. Furthermore, we use the premise that domain-focused searches are a more efficient way to obtain accurate data-trained models.^46^

The choice of using abstracts rather than full articles for end point extraction was based on the fact that abstracts tend to describe the nanomaterial tested, its physicochemical properties, and the numerical result of the toxicological assay performed in each article. These parameters presented in abstracts are strictly related to the results obtained in that specific article. There is a very low probability that results from other studies are mentioned in the abstract for purposes of comparison (this normally occurs in the discussion part of full articles). This aspect leads to a more accurate parameter extraction performed at the final step of the NLP pipeline adopted here. Furthermore, the basic document unit used for processing was the sentences present in the abstracts. We have previous results (not shown here) that showed that LLMs are more efficient at extracting end points like nanomaterial size and concentration at the sentence level than at paragraph level (i.e., feeding the model with a sentence instead of a whole paragraph). In this sense, we made great effort to accurately classify and select sentences that contain the parameters that we want to be extracted. Finally, abstracts presented in indexing databases (e.g., WOS) are more uniformly formatted in terms of Unicode characters.

After downloading the abstracts, a set of REGEX patterns was used to filter the text to correct typos and standardize chemical formulas, physical unity, and others. Examples of REGEX filters include the standardization of physical units (for example, M → mol L-1), chemical formulas (e.g., silicon dioxide → SiO2), and characters (e.g., μ → u). The complete set of REGEX filters is available in the aRIX repository (/Modules/functions_TEXTS.py) on Github.^49^ During text filtering, sentences are split (N = total number of sentences) and isolated for each abstract of the corpus (R = total number of abstracts), indexed with a unique number, and finally structured in CSV (comma separated values) dataframes. A set of unique 1gram-type tokens (for example, words, expressions, punctuation marks, numbers) is then identified for the whole corpus using built-in Python functions (M = number of unique tokens in the corpus). Document-term-type matrices (one-hot, TFIDF) are then generated to feed statistical and machine learning methods that will be used for sentence and abstract classification, such as latent semantic analysis (LSA), latent Dirichlet allocation (LDA), and word vectors (WVs). Details about each of these steps were described by our group elsewhere.^50^ The final list of indexed articles (with DOI) that was included in our corpus is in file “filename_id.csv on Github”.^51^

### Sentences Selection

For this work, we have updated the aRIX program,^49,50^ especially regarding classification and extraction tasks. The program was used here to recognize relevant sentences in the corpus through a concomitant-interchangeable use of (i) REGEX pattern recognition scripts (Lm) and (ii) Semantic (Sm) and (ii) Topic (Tm) machines. Sm identifies sentences in which tokens related to a subject are present. It works when tokens related to this specific subject are provided to the program. The semantic similarity of the tokens is evaluated with the cosine values between the corresponding word vectors of these tokens, which were generated using the Word2Vec approach.^52^ For example, Sm was very useful in determining hundreds of biological species present in corpus by providing just a few samples of species, such as *E. coli*, *S. aureus*, and *B. subtilis*. On the other hand, Tm scans the document-topic-type matrices obtained using the LSA and LDA methods to select sentences (documents) that fit a certain topic (subject).^53,54^ The sentence match during scans occurs when there is a high cosine value between a document-topic vector (a line in the document-topic matrix) and a chosen topic vector T_i_ with dimension *d* (here we used 100). A specific T_i_ vector related to a subject can be generated by summing and normalizing the token-topic vectors of tokens associated with that particular subject. For example, to generate a topic vector for the subject “species used in nanotoxicological assays”, we can sum and normalize the token-topic vectors for *E. coli*, *S. aureus*, and *Daphnia similis* (these vectors are from the token-topic matrix). For details on how document-topic and document-token matrices are generated via LSA and LDA methods, see the Supporting Information.

The purpose of search and extract (SE) routines is to select sentences that contain relevant information to be extracted.^51^ For example, for the SE routine under the tag “nps_size_01”, the program is set to scan the entire corpus of plain text converted from the WOS report (“file_type” = “txt”). The aimed parameter is the size of nanomaterials (defined “nanomaterial_size” as input to field “parameter_to_extract”). The output results will be saved with the filename “nps_size_01”. Because “index_list_name” is “None”, no pre-existing list is used, so every article in the corpus will be scanned. The program operates at the sentence level (”scan_sent_by_sent” = “True”), and repeated values are filtered out (”filter_unique_results” = “True”). No direct literal or semantic entries are provided (”literal_entry” = “None”, “semantic_entry” = “None”), and there is no token-by-token matching or lowercase transformation (”search_token_by_token” = “False”, “lower_sentence_for_semantic” = “False”). In addition, no topic- or section-based searches are employed. Finally, the “num_param” field is set to “nanomaterial_size” to instruct the program to look for numerical expressions that match or are associated with this parameter (e.g., units like “nm”, “*μ*m”).

When categorical parameters are aimed, we can use a set of inputs such as those defined in “metallic_nano_01”. In this search routine, the goal was to find sentences that contain the composition of metallic nanomaterials. In this input, the field “parameter_to_extract” is defined as “metallic_materials”, thus setting the program to extract terms related to metallic elements (e.g., silver, gold, copper). A key difference is introduced in the “semantic_entry”, where the search will focus on the combination of two semantic categories: “metallic_nano_element_name” and “nanomaterial_morphology”. Therefore, sentences containing information on both the composition and morphology of metallic nanomaterials are searched. For details on the term lists related to each category set in the program, see the Supporting Information.

### Parameters Extraction and Data Analysis

The extraction process used the Python library Ollama,^55^ which provides a user-friendly platform for running LLM locally. The LLM selected was mistral-large:123b,^56^ which was chosen based on its superior precision in previous evaluations made by our group on extraction tasks (results not shown). Extensive examples were provided to the model as the role of the system, clarifying the intended function of the model and the expected format of its responses. This approach included both positive examples, i.e., phrases containing the features of interest (e.g., the size of a nanomaterial), and negative examples, i.e., phrases without these features. In cases where no relevant features were identified, the model was configured to respond with “None”. The prompts used to extract each parameter are on file “/Modules/LLM.py”.^49^ The inference parameters temperature, top-p, and top-k were adjusted to optimize the model output for this extraction task. These parameters influence the creativity and variability of model responses by controlling the probability distribution during token generation. For example, as temperature controls randomness, a lower value means more predictable outputs, while a higher value allows for more creativity. Top-p (nucleus sampling) constrains the tokens’ pool to the smallest set whose cumulative probability meets a defined threshold. Similarly, top-k limits the tokens pool to the k most likely candidates. In this study, the values used were 0.3 for temperature, 0.3 for top-p, and 5–50 for top-k (default for mistral-large:123b). Given the goal of extracting the explicit numerical and categorical parameters in sentences, these parameters were set to reduce the generative behavior of the model. This ensured that the responses adhered closely to the input data, avoiding the generation of unexpected responses. After all extractions, all instances were submitted to an LLM filter in verify to determine if where the extracted parameters are indeed correlated. For example, we checked if nanomaterial “A” with properties “P_A, 1_”, “P_A, 2_”, and “P_A, 3_” was tested against species “S” for the end point “E” and that the resulting values “V_A, S, E, 1_”, “V_A, S, E, 2_” and “V_A, S, E, 3_” result from the interaction between “A” and “S” for end point “E”. For this checking, we provided the LLM model with some basic ontology about the entities in nanotoxicology (see section Extraction accuracy test in the Supporting Information).

Possible toxic effects of nanomaterials against biological species were also evaluated in sentences that did not contain numerical end points (e.g., minimum inhibitory concentration - MIC, or lethal concentration 50% - LC_50_). For these cases, we used specialized LLM prompts designed to identify evidence of toxicity in multiple species such as *D. magna*, *D. rerio*, *C. elegans*, and *A. thaliana*. These prompts specifically instruct the LLM to look for indications of categories of adverse effects against these species. Examples of end points include morphological abnormalities, developmental alterations, bioaccumulation, reproductive impairment, and others. Under these guidelines, the model (i.e., mistral-large:123b) parses each sentence for explicit or implicit signs of toxicity against the biological species. If there is clear mention of detrimental results, the model outputs “yes” for “possible toxic effects”. If evidence of a toxic effect is explicitly absent or the sentence states a lack of harmful effects (e.g., “no observed effect”, “not toxic”), the model outputs “no”. If the sentence does not discuss toxicity or cannot be assessed in this context, the model outputs “None”. After the first evaluation of the model, a second prompt is used to confirm whether the previous answer was indeed correct, considering a certain species and a certain end point. This classification process ensures a focused and standardized approach to detect nanotoxicological impacts at the sentence level, facilitating subsequent data aggregation and interpretation. The prompts used are available in file “/Modules/LLM.py”.^49^ With this double-check prompt approach used in the final stage of the extraction pipeline, we achieved a minimum accuracy in extraction of 94%, considering both categorical and numerical parameters (see section Extraction accuracy test in the Supporting Information).

Each SE routine configured in the program generates a CSV file containing the values of the extracted parameters (numerical or categorical). The CSV files are then consolidated into a single CSV file (see files “DFs_to_consolidate*.csv” on Github^51^). To analyze the resulting data frame, the following Python libraries are used: (i) pandas for handling CSV input and data manipulation, (ii) NumPy for numeric array operations, (iii) regex for pattern matching within strings, (iv) Matplotlib along with Seaborn for data visualization, and (v) scikit-learn and (vi) scipy. The general process involves reading the data set into a pandas DataFrame, cleaning the data by removing missing values and duplicates, and grouping or filtering categories according to user-defined criteria. A set of plotting functions is then used to represent the data in different ways.

The final data-frames^51^ include columns for the nanomaterial core composition, nanomaterial morphology, organism species, toxicity end point, and toxicity confirmation, all of which are categorical and do not have physical units. It also contains the nanomaterial size measured in nm, the nanomaterial surface area measured in m^2^ g^–1^, and the nanomaterial zeta potential measured in mV. Additional columns include microbial log reduction, which is dimensionless; microbial minimum inhibitory concentration (MIC) in mg mL^–1^; microbial minimum bactericidal concentration (MBC) in mg mL^–1^; biofilm killing percentage given as percentage (%); and lethal concentration 50% (LC_50_) in mg mL^–1^. The final data frames had a sparsity of around 75% (percentage of zero cells in the matrix).

For statistical analyzes, the results in the data frame could be grouped with categorical labels (e.g., Ag, Au, and SiO_2_ - composition; *E. coli* and *S. aureus* - species) for which there are correlated numerical values (e.g., size, surface area, MIC, MBC). In our observations, these groups have sample sizes, distribution shapes, and variances that are markedly different, rendering the equal-variance assumption of the classical one-way *F*-test untenable. For statistical comparisons, we therefore adopted Welch’s *F* test, an adaptation of the classical ANOVA statistic that explicitly adjusts the degrees of freedom of the denominator and is therefore especially robust when the assumption of homoscedasticity is violated. In the global comparison among several groups, we embedded the Welch (heteroscedastic) *F*-statistic in a permutation frame: the observed values were pooled and their group labels were randomly reassigned at least 20 000 times while preserving the original group sizes, and the statistic (i.e., 10% trimmed mean) was recalculated after every relabeling. The proportion of permuted values that exceeded the observed value supplied an exact permutation *p*-value for the omnibus null hypothesis of equal means across all groups.

Because no single omnibus test can reveal which specific pairs of groups differ, we next examined every one of the possible pairs. For each pair, we used Welch-studentized mean difference. The procedure is distribution-free: we computed the observed difference in the trimmed means (10% symmetric trimmed), permuted the two group labels at least 20 000 times, recomputed the statistic on each permutation, and obtained pairwise *p*-value from the resulting null distribution. We chose to inspect all pairs irrespective of the global test result. A nonsignificant global test can conceal a single large contrast when variance is high, so any significant pair emerging in that context must be interpreted with caution, especially with small or highly variable samples. Finally, trimmed means (10% symmetric trim) were used as the standard statistic here because the numerical values (e.g., size, zeta potential, MIC, MBC) have a natural interpretation and extreme observations can carry a genuine informational weight.

On the choice of the statistical test, we used the permutation-based version of Welch’s test rather than a nonparametric test such as Kruskal–Wallis mainly because our group sizes were quite unequal and varied significantly. Welch’s test is known to be robust to unequal variances and sample sizes, and by applying a permutation approach, we further ensured that our inference did not rely on normality assumptions. In other words, this choice allowed us to handle both the unequal group sizes and any potential distributional issues. In this way, we believe that this approach provided a robust statistical framework that suited the structure of our data.

## Results

### Corpus Analysis

When aRIX set the search and extraction routines, we sought to assess the types of nanomaterials, exposure methods, observed toxicological effects, and experimental models used in the literature. The strategy taken to identify nanotoxicology articles in WOS was to combine a wide range of terms related to the nanomaterial morphology with the most common biological organisms studied (see the Supporting Information for details). By including both common and scientific names of biological species, as well as abbreviations, we sought to increase our retrieval. This approach ensured that articles using different nomenclature were captured (for instance, searching for both “*E. coli*” and “*Escherichia coli*”). In addition, conducting a combined search using terms related to nanomaterial morphology and biological organism species is more precise than using a general term such as “nanotoxicology”. Nanotoxicology is an emerging multidisciplinary area that intersects various fields, including chemistry, biology, and environmental science, and researchers in this area may describe specifically nanomaterials or organisms without using the term “nanotoxicology”.

The topic modeling of the corpus was performed both via Latent Semantic Analysis (LSA) and Latent Dirichlet Allocation (LDA), at the sentence and abstract level. Graphical representations of the resulting token topic matrices indicated a better performance of the LDA method to separate documents and tokens into possible topics (see Figure S1 in the Supporting Information). The token vectors obtained via the LDA method are more homogeneously spread over the representation space in comparison to those obtained via LSA, for the modeling both at sentence and abstract level. The LSA approach was successful in identifying only a few major topics related to the production and application of nanomaterials (e.g., Ag-based, TiO_2_, ZnO), especially as antimicrobial agents (see Tables S3 and S4 in the Supporting Information). Other topics with fewer occurrences in the corpus could not be accurately identified through this approach, both at the abstract and sentence level.

The topic modeling for abstracts via the LDA approach (see Table S5 in the Supporting Information) was capable of separating articles considering many topics, such as (i) Bacterial Infections, Antibiotic Resistance and Pathogen Detection in Nanotoxicology; (ii) Photocatalytic Nanomaterials for Visible-Light Applications; (iii) Environmental Nanotoxicology and Ecotoxicological Assessments; (iv) Nanomaterial-Based Wound Healing and Tissue Regeneration; (v) Advanced Photocatalytic Inactivation and Water Disinfection; (vi) Toxicological Impacts of Nanomaterials on Aquatic Organisms; and (vii) Antimicrobial Testing and Food Safety Monitoring with Nanomaterials. Despite the successful identification of many topics, we observed a large overlap among topics at the abstract level (see Figure S1b). However, the topic modeling for sentences via LDA was much more efficient in separating several subjects described at the sentence level (see Figure S1d and Table S6 in the Supporting Information).

Among the results of the LDA topic modeling for sentences (100 possible topics), sentences related to the production, characterization, application, and impacts of AgNPs were largely captured in the topics. There are sentences related to (i) the green synthesis of AgNPs using plant extracts as reducing agents. This involves methods of preparing AgNPs by extracting compounds (e.g., leaves) that help reduce metal ions in an environmentally friendly and green process. The following sentences contain information on (ii) the characterization of AgNPs and other nanomaterials using multiple analytical techniques: X-ray diffraction (XRD), scanning electron microscopy (SEM), transmission electron microscopy (TEM), Fourier transform infrared (FTIR) spectroscopy, and UV–vis spectroscopy to analyze their structure, morphology, and composition. Another relevant topic was related to sentences describing (iii) the evaluation of antibacterial efficacy (e.g., through the minimum inhibitory concentration, MIC) of AgNP against bacteria such as *E. coli* and *S. aureus*. This involves measuring concentrations in μg mL^–1^ or mg mL^–1^ and determining inhibitory and bactericidal effects. (iv) Sentences with applications of AgNPs in antibacterial wound dressings were also classified.

Considering other nanomaterials, the LDA topic modeling captured sentences related to the (v) synthesis, characterization, and surface functionalization of CNTs - both single-walled (SWCNT) and multiwalled (MWCNT) - potentially including their integration with TiO_2_ or other materials for advanced nanotechnological applications. The description of (vi) type II quantum dots (QDs) or related nanoparticles for fluorescence-based studies of protein binding and metal ion interactions in biological systems is also given in the following sentences. The tokens suggest research on how these QDs interact with proteins and cells, particularly with regard to fluorescence detection/imaging and potentially comparing wild-type vs modified proteins or cells. In terms of toxicity assays, there are topics related to the investigation of the potential toxicity and environmental impact of nanoparticles on multiple organisms, assessing how nanomaterials (particularly in natural and organic contexts) may affect the environment. More specifically, there are sentences describing (vii) the acute evaluation of toxicity of titanium dioxide (TiO_2_) nanoparticles on aquatic organisms (e.g., *D. magna* and *Chlorella vulgaris*), investigating different concentrations and their potential effects on freshwater ecosystems; (viii) the evaluation of nanoparticle-induced toxicity in the nematode *C. elegans*, focusing on oxidative stress, impacts on growth and reproduction, and organism stress response mechanisms; (ix) the tests of nanoparticles on zebrafish (*D. rerio*) embryos, assessing mortality rates (LC_50_ values) at varying concentrations (mg L^–1^, μg L^–1^) over specific exposure times (h); (x) the tests of titanium dioxide (TiO_2_) nanoparticles on zebrafish embryos, focusing on developmental effects, exposure outcomes, and overall toxicity in a model organism; and (xi) the development and evaluation of silver (Ag) and zinc oxide (ZnO) nanoparticle-based nanocomposites with enhanced antibacterial (antimicrobial) properties.

It is important to mention that using 100 topics can introduce some overlap or redundancy in the identified topics. However, our intention in opting for a relatively large number of topics was to achieve a finer granularity. This allows us to pinpoint specific sentences more accurately, especially when dealing with a diverse range of nanomaterials and biological contexts. To illustrate this aspect, we include a hierarchical clustering graph of the topics (see Figure S2 in the Supporting Information). Although some redundancy is present, this approach ensures that we can model the distribution of topics at the sentence level, thereby enhancing the precision of information extraction. In other words, the choice of a larger number of topics was a deliberate trade-off to achieve a more detailed and fine-grained representation of topics, since only a few topics had redundancy (see T95-T13-T46; T72-T74; T89-T52; T43-T10-T49; and T65-T86 in Figure S2).

In Figure 1a, the horizontal bar charts show the frequency of occurrence of the composition and morphology of nanomaterials in the corpus. Silver (Ag), as the most studied composition with 20,168 occurrences, was extensively introduced into nanotechnological products due to its antimicrobial applications. Zinc Oxide (ZnO) and Gold (Au) follow with 7,461 and 3,783 occurrences in our corpus, respectively, reflecting their applications in cosmetics, electronics, and in the medical area. Carbon-based materials such as graphene, carbon dots (CDots) and carbon nanotubes (CNTs) were also studied considering their possible interactions with biological systems, along with copper (Cu, metallic and oxide form), ion (Fe, metallic and oxide form), silicon dioxide (SiO_2_) and titanium oxide (TiO_2_). In the biological organisms studied ([Fig fig1]b), *E. coli* is the species most studied, with 5,593 occurrences, followed by *S. aureus* with 4,599 occurrences. This highlights their prevalence as model organisms for studying nanotoxicological impacts, especially in pathogenic inhibition contexts. Other microorganisms such as *Pseudomonas aeruginosa*, *B. subtilis*, *Klebsiella pneumoniae* and *Candida albicans* were substantially studied in the context of bacterial and fungal toxicity. This result describes a range of species, from bacteria and fungi to invertebrates like *D. magna* and nematodes like *C. elegans*. This diversity highlights interdisciplinary efforts in nanotoxicology. Furthermore, this distribution demonstrates a strong focus on bacterial species, particularly common laboratory models and pathogens. However, the inclusion of other organisms reflects the field’s mission to assess broader ecological and biological impacts of nanomaterials. Finally, when the morphology was analyzed (see Figure S3 in the Supporting Information), it was evident that nanoparticles were present in most studies, with 41,657 occurrences in our corpus. Nanoparticles played a central role in nanotoxicology studies as a result of their broad applications and potential impacts. Other morphologies such as dots, nanotubes, and nanostructures were also relevant in the corpus, while nanorods, nanowires, nanospheres, and others were substantially less studied, potentially representing areas for future research in the field.

**Figure 1:**
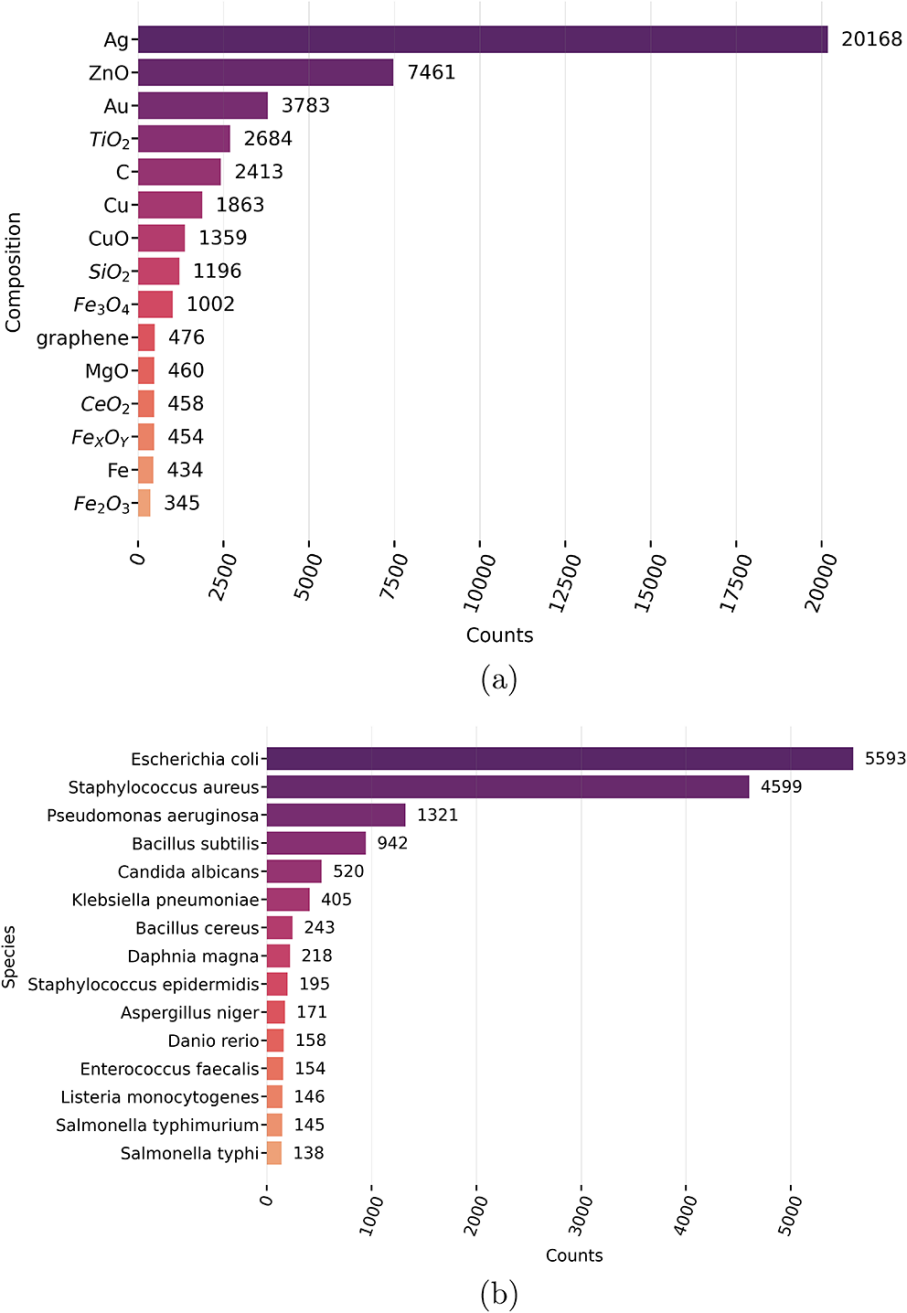
Frequencies (counts) of terms occurring in the nanotoxicology corpus analyzed. Horizontal bar-plots describe occurrences of terms related to (a) nanomaterials composition and (b) organisms species.

### Nanomaterials and Their Characteristics

The nanoparticle is the morphology most commonly studied in various compositions, as indicated by the heatmap in Figure 2. Materials such as Ag, Au, ZnO and TiO_2_ have been well studied in multiple morphologies (e.g., nanoparticles, nanotubes, and nanorods), representing their significant role in nanomaterial applications and potential toxicity. Unique morphologies are described for CdSe (dots) and MoS_2_ (nanosheets). Most of the articles in the corpus used nanomaterials in the 1–100 nm range, and the sizes related to the top 16 composition categories are shown in Figure 3a. The mean size of nanomaterials was greater than 20 nm, with the exception of some quantum dots (CdS, CdSe, ZnS) and some metallic nanoparticles (Al, Pd, Pt; see Table S7 in the Supporting Information). The size distributions varied greatly with the composition of the nanomaterials. Welch F test for a global comparison between the compositions indicated a statistical difference among them (*p*-value <0.01). A pairwise comparison using the Welch-studentised mean difference (with 20,000 permutations) between the compositions indicated which of them were statistically different (see Figure S4a in the Supporting Information). Among the compositions studied the most frequently, we see that Ag nanomaterials were statistically smaller than Cu, CuO, SiO_2_, TiO_2_ and ZnO. SiO_2_ nanomaterials tested were larger than most of the other compositions. In terms of the zeta potential (ζ) of nanomaterial dispersions, the mean values ζ were found to be negative for all compositions identified, with a global statistical difference between them (*p*-value <0.01; Figure 3b and Table S7 in Supporting Information).

**Figure 2:**
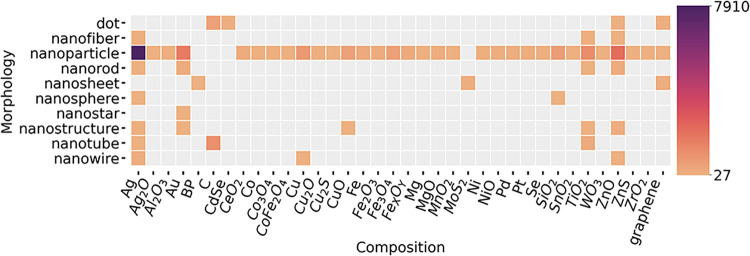
Frequency distribution (counts) of combined terms occurring in the nanotoxicology corpus analyzed. The correlations between the nanomaterial morphologies and the nanomaterial composition were assessed. The color scale represents the occurrence counting, with darker shades indicating higher frequencies.

**Figure 3:**
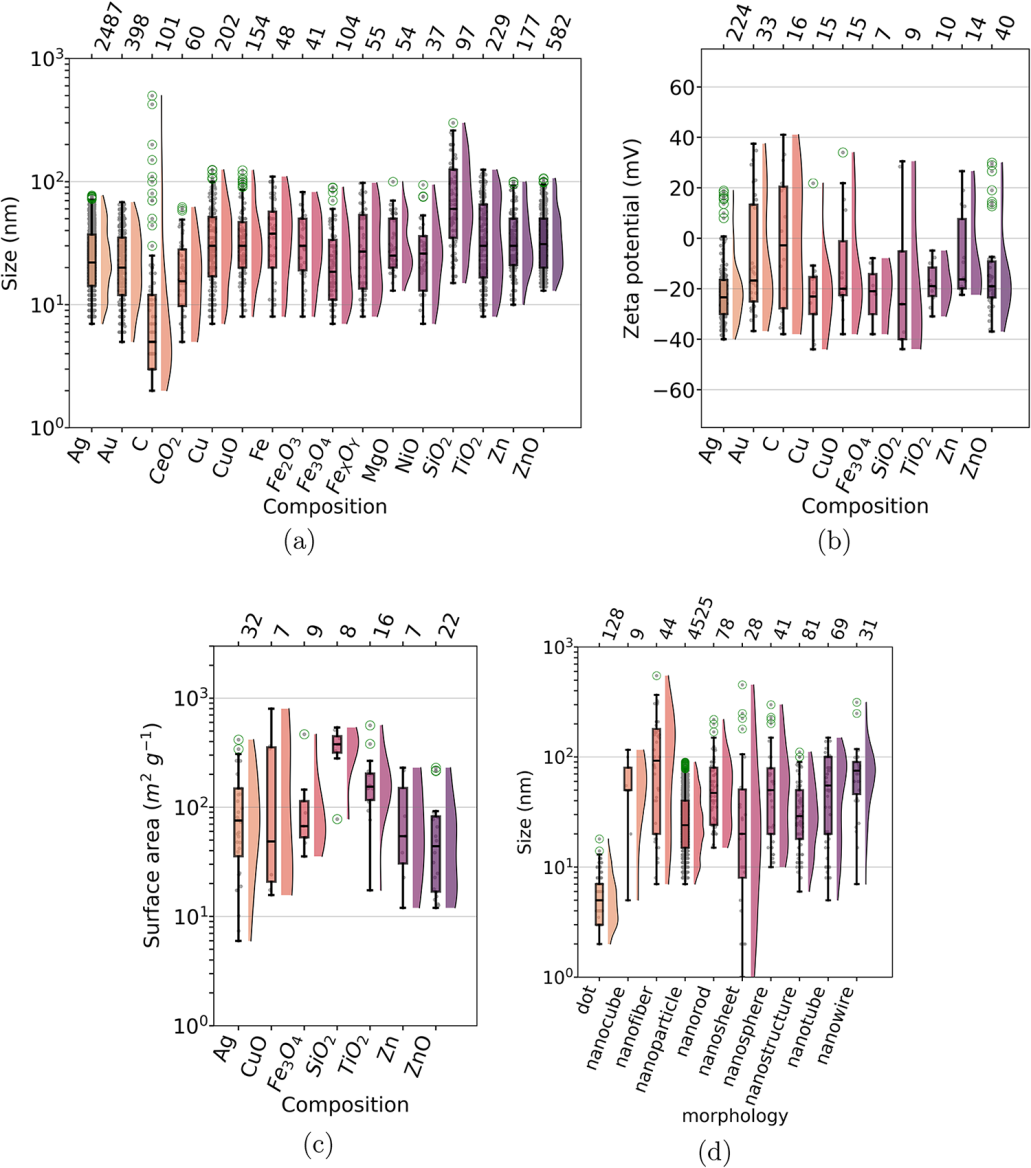
Box-plot graphs representing relations between categorical and numerical parameters extracted from the nanotoxicology corpus. (a) size, (b) zeta potential and (c) surface area distributions of nanomaterials as a function of the composition, and (d) size distribution as a function of the nanomaterials morphology. Numbers at the top of the box-plots indicate how many articles were used in each values distribution. Box-plots were plotted for categories for which at least 7 numerical values were extracted. The distribution function found via kernel density estimator (KDE) for each box-plot is represented at the right side.

When considering surface area as a function of the composition of nanomaterials, significant differences were observed (Welch F test p-value <0.01; see Figures 3c and S4c, shown in the Supporting Information). The tested silica nanomaterials had a substantially larger surface area (see Table S7 in the Supporting Information) compared to nanomaterials with a base composition of Ag and ZnO, for example. This may be related to biological tests performed with mesoporous silica, which is considered a nanocarrier in many technological applications. Au is one of the most tested compositions in the corpus, but few articles describe the surface area of the dried nanomaterial. In addition, significant differences were also found when comparing the size distributions between the nanomaterial morphologies (*p*-value <0.01; Figure 3d). The dots have a smaller size compared to all other morphologies (see Table S8 in the Supporting Information). The mean size of the tested nanoparticles was smaller than that of other morphologies such as nanofibers, nanorods, nanospheres, nanotubes, and nanowires (see Figure S4d in the Supporting Information). Finally, the surface area described for the nanomaterials with different morphologies tested did not show significant differences between the morphologies (see Table S8 in the Supporting Information).

Most nanomaterials dealt in the corpus exhibit negative zeta potentials throughout the size range of ≈ 1–10^3^ nm (see Figure S5a in the Supporting Information). Positively charged particles are comparatively rare in the corpus; thus the evidence base is biased toward anionic/near-neutral materials. We also note that the zeta potential is medium-dependent (pH, ionic strength, dispersants) and that the reported sizes mix DLS and microscopy metrics. In the size-surface-area scatter plot (see Figure S5b in the Supporting Information), the highest surface areas cluster among sub-10 nm particles, consistent with the expected geometric scaling for nonporous particles. However, above ≈ 10 nm, the surface area values become highly variable, spanning approximately 1 to 10^3^ m^2^ g^–1^. This dispersion likely reflects heterogeneity in morphology and porosity (e.g., meso/microporous oxides and carbons, platelets, and nanosheets). In particular, particles in the 10–100 nm range can exhibit substantial internal or accessible porosity, which increases the surface area of BET independently of the external size. Finally, very few paired observations between surface area and zeta potential were extracted (see Figure S5a in the Supporting Information). Importantly, these two parameters are governed by different factors: the zeta potential depends on the chemistry of the surface and the medium conditions (pH, ionic strength, and dispersants), whereas the specific surface area reflects morphology and porosity. We interpret the sparsity as a reporting gap (few studies report both together) and highlight the need for standardized, paired reporting and stratified analyses by material class/functionalization to allow robust multivariate evaluation.

### Interactions with Microorganisms

In the analysis of the different species exposed to nanomaterials (Figure 4), we observed that the studies cover a range of organisms, from bacteria (*B. subtilis*, *Streptococcus mutans*) to fungi (*C. albicans*, *Aspergillus flavus*) and eukaryotic models (*C. elegans*, *D. magna*). *E. coli* has the highest frequency in multiple compositions, particularly for Ag, Au, C, Cu, SiO_2_, TiO_2_, and ZnO. The results indicate the common use of *E. coli* as a model organism in nanotoxicology studies due to its simplicity and relevance in microbiological research. In addition, *S. aureus* also shows significant importance in nanotoxicological studies for these same compositions, possibly because of its role in pathogenic studies. Fungi (*C. albicans*), invertebrates (*D. magna*), and vertebrates (*D. rerio*) were also studied, although more restricted to certain compositions (especially Ag). Taking into account the correlation between organism species and nanomaterial morphologies, *E. coli* and *S. aureus* were the most studied in all morphologies (see Figure S6 in the Supporting Information). Nonmicrobial species such as *D. rerio* (zebrafish) and *A. thaliana* are less represented, probably due to their more specialized use in ecotoxicology and plant toxicity studies.

**Figure 4:**
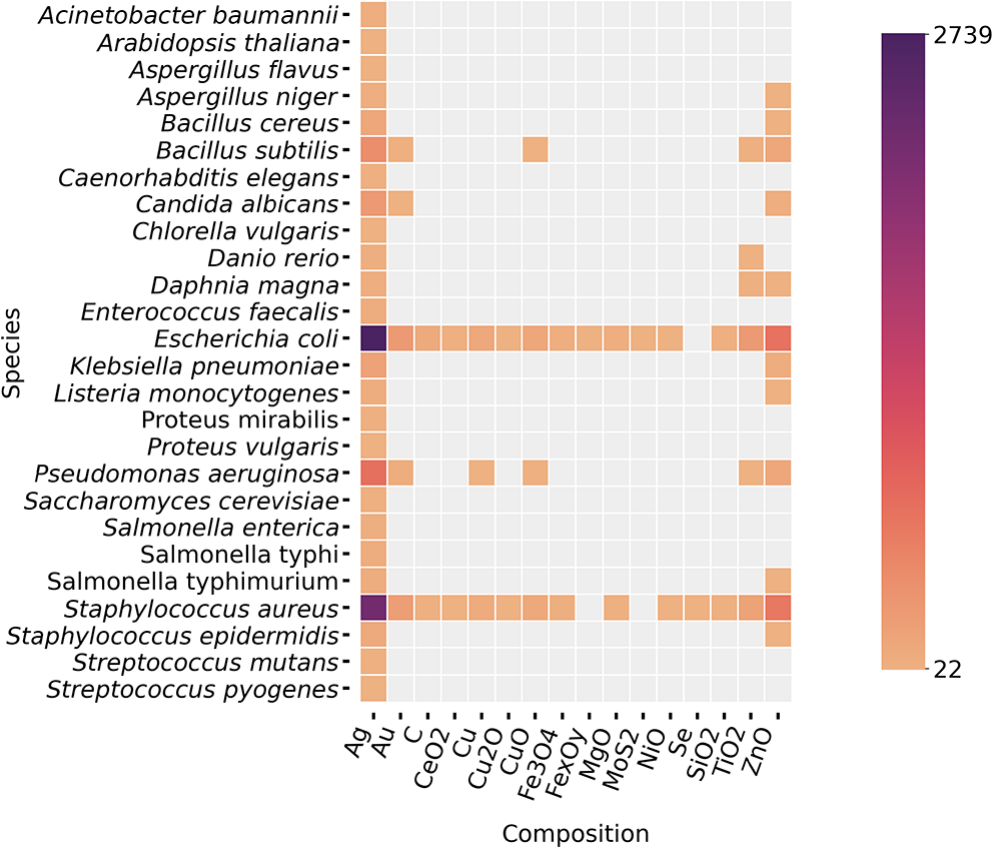
Frequency distribution (counts) of combined terms occurring in the nanotoxicology corpus analyzed. The correlations between the nanomaterial composition and the organism species tested were assessed. The color scale represents the occurrence counting, with darker shades indicating higher frequencies.

Due to the large use of microorganisms in biological assays described in the corpus, some numerical parameters (end points) are strictly related to the evaluation of antimicrobial efficacy. Logarithmic reduction represents a way to express the reduction in the number of microorganisms after treatment with nanomaterials. A 1-log reduction corresponds to a 90% decrease in microbial count, while a 2-log reduction corresponds to a 99% decrease. In terms of the composition of the nanomaterials, we observed a difference among the Ag, Au, Cu, TiO_2_ and ZnO-based nanomaterials (*p*-value <0.05). Mean log reduction values found for Ag and Cu nanomaterials were higher than the others (see Figure S7a and S7b). More specifically, Ag nanomaterials were higher than those of Au, TiO_2_ and ZnO (*p*-values <0.05; see Table S7). However, when we filtered the log reduction values obtained just for *E. coli* no statistical difference was found. Finally, no statistical differences was observed in logarithmic reduction for the different morphologies of the nanomaterials (S8).

When trying to establish log-reduction comparisons for different microorganisms considering just one composition (e.g., Ag) or morphology (e.g., nanoparticle), there was not a sufficient amount of data extracted for this specific end point. However, the minimum inhibitory concentration (MIC) was widely evaluated as an end point in the evaluation of antimicrobial effects. MIC represents the lowest concentration of a nanomaterial that prevents visible growth of a microorganism (such as bacteria). When analyzing the MIC results, we observed significant differences between compositions (*p*-values <0.01; see Figures 5a, S8a and Table S7). In general, Ag-based nanoparticles had the lowest median value among the groups, while the mean value was higher due to a number of outlier values for their distribution.

**Figure 5:**
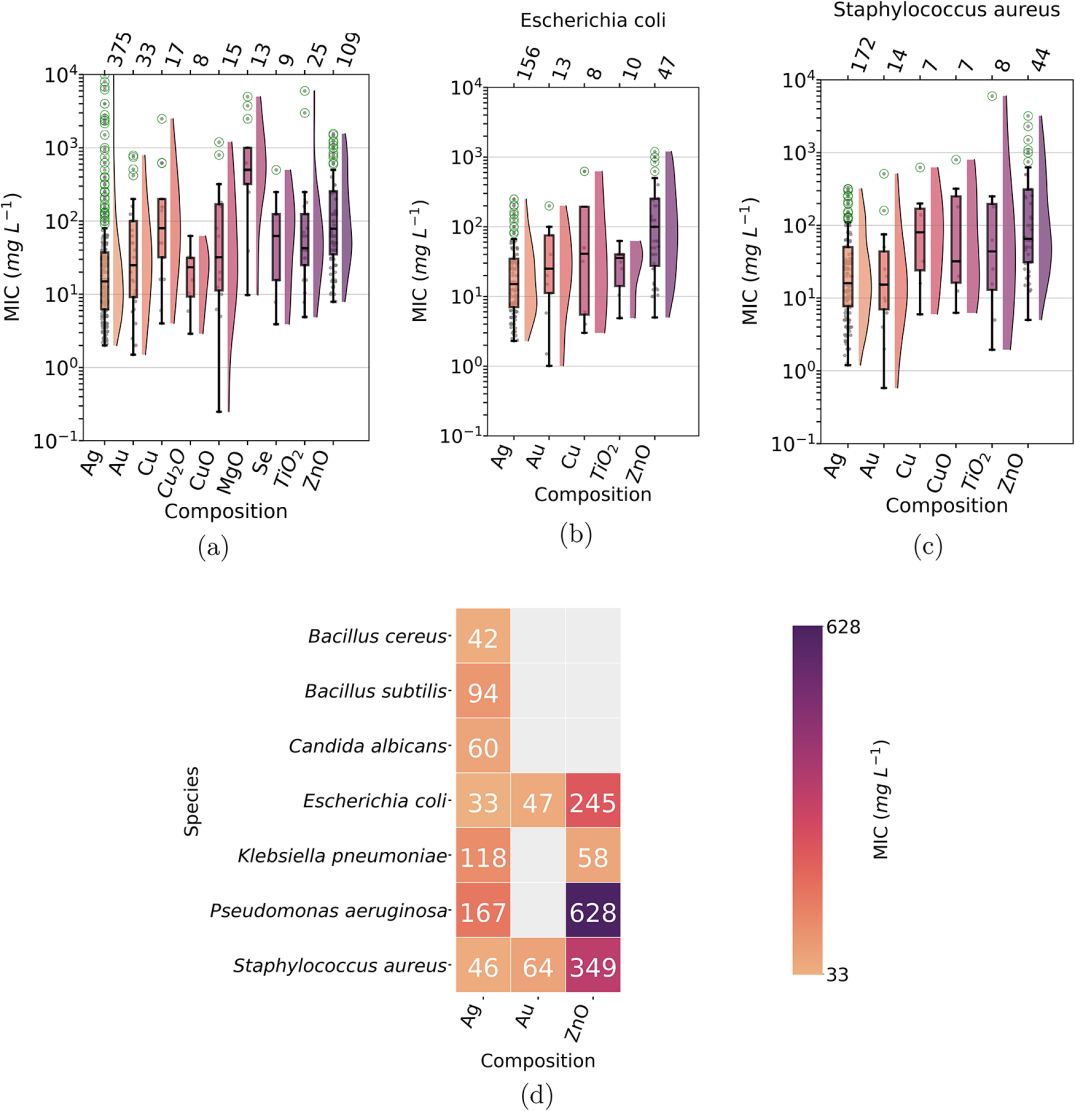
Box-plot graphs representing relations between categorical and numerical parameters extracted from the nanotoxicology corpus. (a) Minimum inhibitory concentration (MIC) as a function of the nanomaterial composition. The graphs can be filter to represent values related only with certain organisms species such as (b) *E. coli* and (c) *S. aureus*. Numbers at the top of the box-plots indicate how many articles were used in each values distribution. Box-plots were plotted for categories for which at least 7 numerical values were extracted. The distribution function found via kernel density estimator (KDE) for each box-plot is represented at the right side. (d) Heatmap showing mean MIC values as a function of the nanomaterials composition and the biological species tested. The colors in the heatmap and the number inside the boxes indicate the mean MIC values.

A more relevant analysis can be done by filtering just the results obtained for a determined biological species. Taking into account only *E. coli* (Figure 5b), we observed that Ag nanomaterials have low MIC values and are statistically different from ZnO (*p*-value <0.05; see Figure S8b and Table S9 in the Supporting Information). In comparison, ZnO nanomaterials have higher MIC values than those of Au and TiO_2_ (*p*-value <0.05). A similar result was found when only antimicrobial tests against *S. aureus* were considered (Figure 5c). Against *S. aureus*, Au and Ag nanomaterials have low MIC values. Furthermore, ZnO had higher MIC values compared to Au and Ag (*p*-value <0.05; see Figure S8c in the Supporting Information). We interpret these pair comparisons with caution since the global Welch *p*-value for MIC values against *E. coli* and *S. aureus* was 0.16 and 0.34, respectively. However, other end points discussed below will confirm these differences.

We consider the tests of Ag-based nanomaterials against other microorganism species such as *Bacillus cereus*, *B. subtilis*, *C. albicans*, *K. pneumoniae* and *P. aeruginosa*, the mean MIC values were in the range of 30–170 mg L^–1^ (Figure 5d). In addition, if we consider only the median values, Ag nanomaterials have lower MIC values compared to other compositions, against several microorganisms (see Table S9 in the Supporting Information).

It is also possible to correlate the size of the nanomaterials with the MIC values. Taking into account all extracted values, we observed that the data points are densely concentrated below 100 nm, particularly below 50 nm, reflecting the typical dimensional range of nanomaterials used in antimicrobial applications (see Figure S9a in the Supporting Information). In terms of MIC, the majority cluster between 1 and 10^2^ mg L^–1^, indicating that many of the nanomaterials tested demonstrate strong antimicrobial properties. Although a strict linear correlation was not observed between the nanomaterial size and MIC, it is noticeable that the most potent nanomaterials, those requiring lower concentrations to inhibit microbial growth, are generally smaller in size. Larger particles appear less frequently and tend to be associated with higher MIC values. Some pairwise comparisons between different core compositions of nanomaterials indicated statistical differences among the materials. For a similar size range, the MIC values of Ag-based nanomaterials were smaller than those of ZnO in tests against both *E. coli* and *S. aureus* (*p*-value <0.05; see Figure 6a,b).

**Figure 6:**
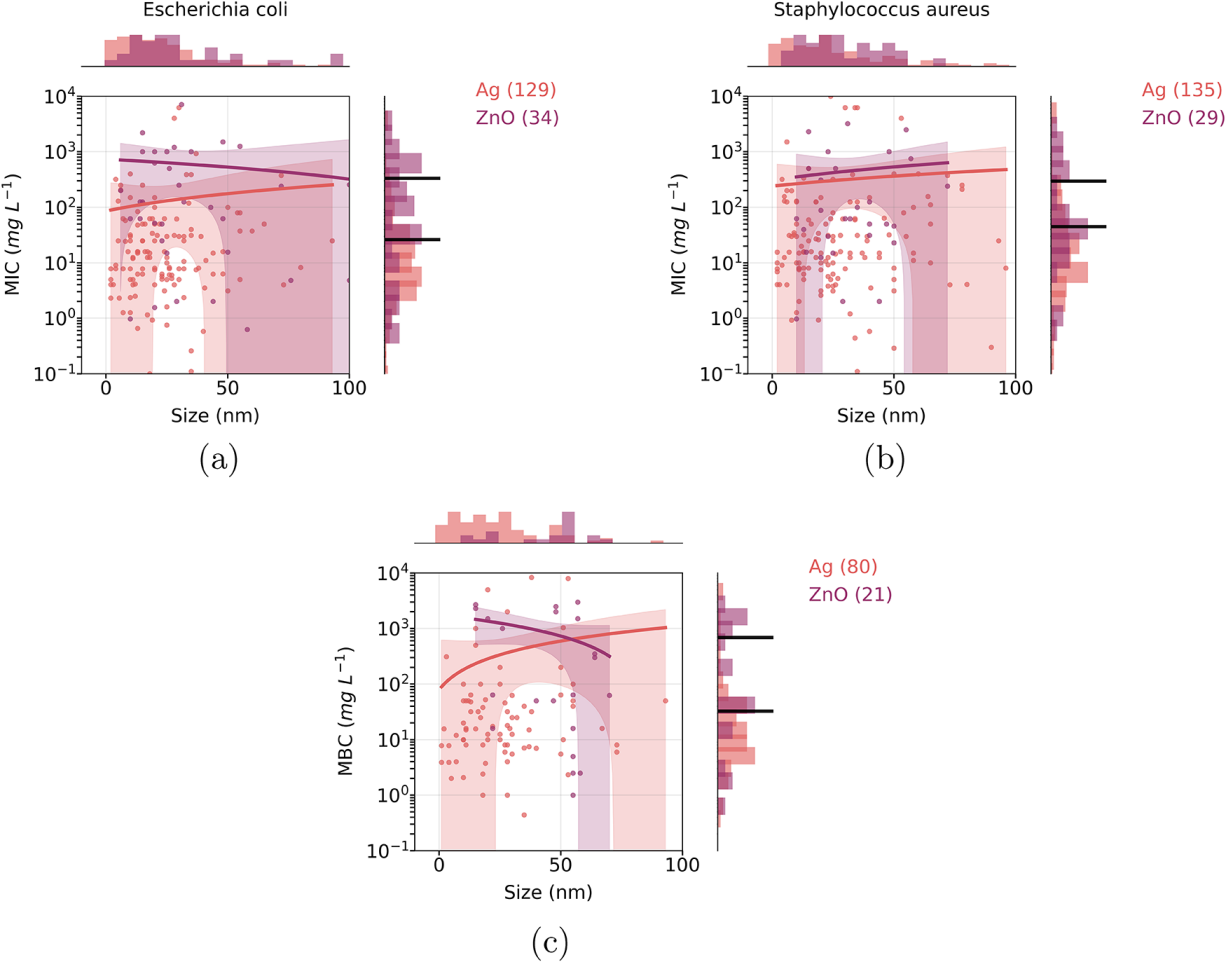
Scatter plots showing the nanomaterials size distributions versus the minimum inhibitory concentration (MIC) for microorganisms (a) *E. coli* and (b) *S. aureus*. A scatter plot is also shown for the minimum bactericidal concentration (MBC) considering all species (c). Histograms representing the values distributions are displayed at the top and right in the graphs. Comparison between distributions were made for most studied compositions (Ag and ZnO). Statistical differences among the distributions are showed the horizontal black lines in the histograms (lines are positioned at the mean value of the distribution). The curve in each panel shows the linear regression fit, and the shaded band indicates the 95% confidence interval (CI).

A similar result was found for another end point, the minimum bactericidal concentration (MBC). MBC is another end point in nanotoxicology assays and represents the lowest concentration of a nanomaterial agent that kills 99.9% of the original bacterial population. Compared to MIC, this end point had fewer occurrences in the corpus. However, for most studied nanomaterials, such as Ag and ZnO, we observed significant differences in their value distributions. MBC for Ag-based nanomaterials tends to be lower than that for ZnO, considering all species present in the corpus (*p*-value <0.05; Figure 6c). In general, the lowest MBC values occurred with nanomaterials in the size range of 1–50 nm (see Figure S9b).

Ag-based nanomaterials also presented an overall higher efficiency against microorganisms in the biofilm state (*p*-values <0.05; see Figures S11a and S11b). This was evaluated using the biofilm killing percentage (BKP) end point. BKP indicates the fraction of microorganisms in a biofilm that are eliminated after exposure to the nanomaterial. Since biofilm-embedded bacteria are generally more resistant than their planktonic counterparts, a high killing percentage (typically above 90%) is often a sign of effective antimicrobial activity. This effect was observed in pairwise comparisons with Au and ZnO nanomaterials.

### Interactions with Other Organisms

In addition to the interactions with microorganisms, we sought end points that would provide information on the interactions of nanomaterials with other species (e.g., crustaceans, fish, nematodes, plants, and insects). LC_50_, or lethal concentration 50%, indicates the concentration at which 50% of a population tested is affected by the nanomaterial. It was extracted LC_50_ values for the interaction of nanomaterials with *Acinetobacter baumannii*, *Aedes aegypti*, *Anopheles stephensi*, *Anopheles subpictus*, *Artemia salina*, *A. flavus*, *Aspergillus fumigatus*, *Bacillus amyloliquefaciens*, *Bacillus cereus*, *B. subtilis*, *Balanus amphitrite*, *Biomphalaria alexandrina*, *C. elegans*, *Ceriodaphnia cornuta*, *Ceriodaphnia dubia*, *Culex pipiens*, *Culex quinquefasciatus*, *Culex tritaeniorhynchus*, *D. rerio*, *Daphnia carinata*, *Daphnia lumholtzi*, *D. magna*, *Daphnia pulex*, *Dunaliella salina*, *E. coli*, *Gobiocypris rarus*, *Hyalella azteca*, *Hydra attenuata*, *Hydra vulgaris*, *K. pneumoniae*, *Lemna valdiviana*, *Litopenaeus vannamei*, *Moina macrocopa*, *Nitellopsis obtusa*, *Oncorhynchus mykiss*, *Oreochromis mossambicus*, *Oreochromis niloticus*, *Poecilia reticulata*, *Proteus mirabilis*, *P. aeruginosa*, *Salmonella typhi*, *Sclerophrys arabica*, *Shigella flexneri*, *S. aureus*, *Xenopus tropicalis*.

Taking into account all these species, the scatter distribution of nanomaterial size versus LC_50_ did not indicate a clear linear or monotonic trend between nanomaterial size and toxicity (see Figure S12 in Supporting Information). Nanomaterials with size smaller than 50 nm show a wide range of LC_50_ values, ranging from highly toxic (10^–1^ mg L^–1^) to moderately toxic (>10 mg L^–1^). Larger nanomaterials, such as those above 50 nm, appear less frequently in the data set and tend to cluster around values of LC_50_ above 10 mg L^–1^, suggesting moderate toxicity. In a comparison between the core composition of the nanomaterials that have the highest number of extracted LC_50_ values, no statistical differences was were observed between Ag and ZnO (results not shown). LC_50_ tests for Ag and ZnO represent most of the extracted instances for this end point.

Due to the variety of other possible end points used in nanotoxicity assays against higher organisms (compared to microorganisms), we performed prompt-specific tasks in the LLM model to confirm the possible toxic effect of a nanomaterial against a specific species. We developed specific prompts for *D. magna*, *D. rerio*, *C. elegans* and *A. thaliana*, which are among the organisms studied the most (see Figure 7). For articles reporting tests with these organisms, all sentences with possible information on the toxic behavior of nanomaterials against these species were provided as input to the LLM model. The ratio between positive (toxic) and negative (not toxic) responses with respect to several end points was analyzed (see Figure S13 in the Supporting Information). Taking into account all biological species reported in the corpus, Ag exhibits a broad spectrum of toxicity, showing high percentages of oxidative stress, photosynthetic impairment, and behavioral impairment. In addition, moderate toxicity has been reported for developmental alterations, morphological abnormalities, and reproductive impairment. TiO_2_ also shows a broad toxicity profile with high responses to oxidative stress, morphological abnormalities, and reproductive impairment. The ZnO nanomaterials had a high level of positive responses to reproductive impairment and consistently with high values for oxidative stress and moderate toxicity with respect to developmental alterations, acute mortality, and genotoxicity. The general level of bioaccumulation reports was low for Ag, C, TiO_2_ and ZnO.

**Figure 7:**
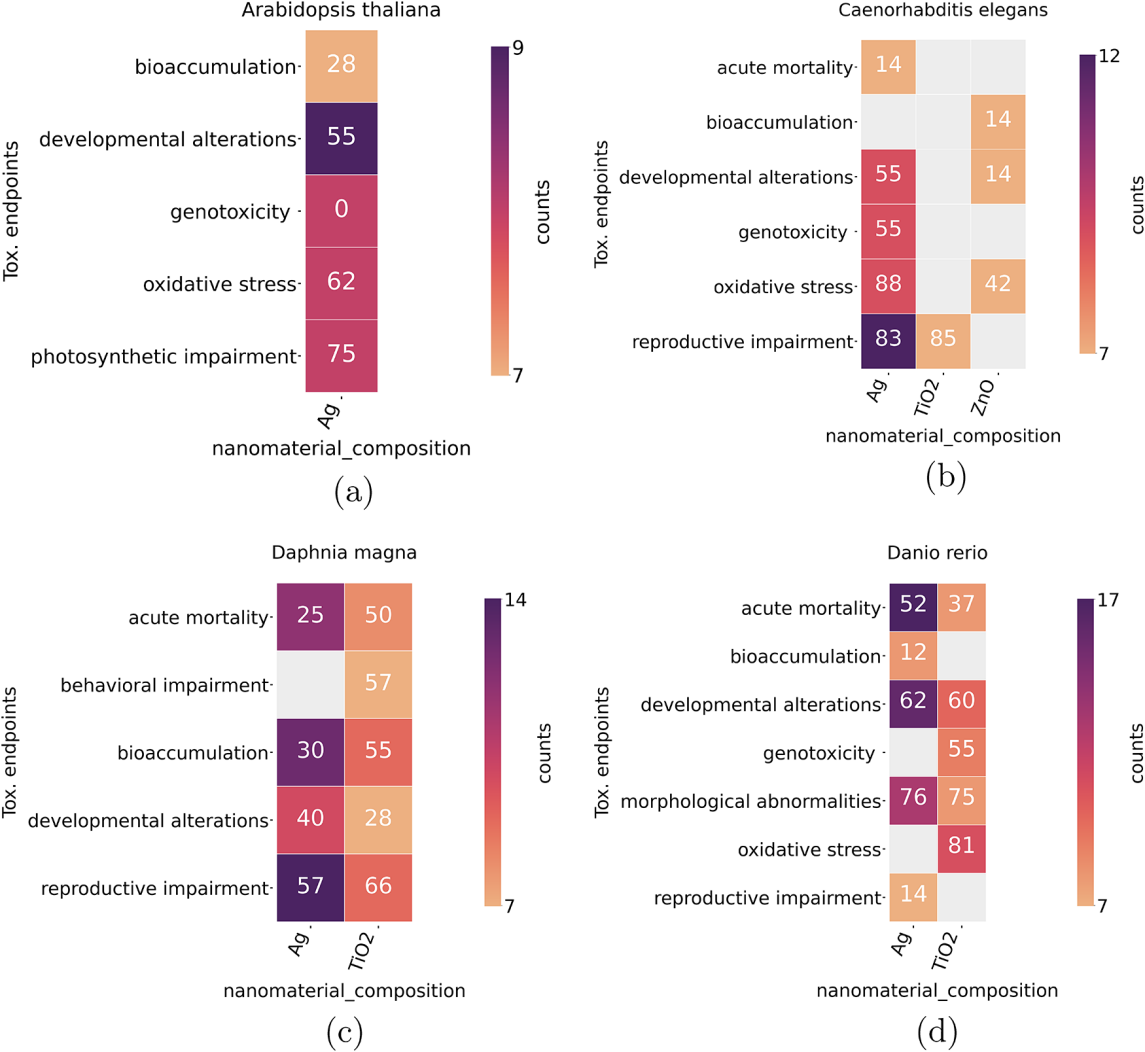
Heatmap showing the percentage of the LLM positive answers when asked about the possible toxicity of nanomaterials considering varied end points. Heatmaps display the nanomaterials composition as a function of the toxicity end points for (a) *A. thaliana*, (b) *C. elegans*, (c) *D. magna*, and (d) *D. rerio*. The colors indicate the number (counts) of sentences that were analyzed by the LLM while the number inside the boxes indicate the percentage of positive answers from the model (LLM). Percentages were calculated when at least 7 sentences were analyzed for a determined nanomaterial composition and a toxicity end point.

When we filtered the answers for *A. thaliana* (Figure 7a), the results show that silver nanoparticles (Ag) predominantly affect plant health through photosynthetic impairment and oxidative stress. These results are consistent with known phytotoxic effects, as nanoparticles often generate reactive oxygen species that interfere with chloroplast function. Developmental alterations were reported with moderate frequency, suggesting some structural or growth-related disturbances. However, bioaccumulation showed that less positive responses toward toxicity and genotoxicity was not detected in current data. For *C. elegans* (Figure 7b), the model identifies oxidative stress as the most prominent toxicological end point, especially with Ag nanoparticles and, to a lesser extent, ZnO. Reproductive impairment also has a high feature for Ag and TiO_2_. These findings point to significant sublethal effects related to reproductive fitness. Genotoxicity and developmental alterations are moderately represented for Ag, but developmental alterations drop for ZnO, indicating material-specific variations in developmental toxicity. The acute mortality report is relatively low, suggesting that while death is not commonly observed, chronic effects on oxidative balance and reproduction are likely.

For *D. magna* (Figure 7c), reproductive impairment stands out as the end point with the most frequent positive toxicity responses. Behavioral impairment is also notable. Bioaccumulation positive answers are moderate, indicating some uptake of particles. Developmental alterations show weaker signals and acute mortality was relatively less reported, again pointing to more chronic or sublethal toxicity pathways rather than immediate lethality. For zebrafish (Figure 7d), the results reveal that oxidative stress is the most consistently detected effect, both for Ag and TiO_2_. Morphological abnormalities and developmental alterations are also frequently mentioned, suggesting that exposure to these nanoparticles during the early stages of life leads to substantial structural and developmental defects. Genotoxicity is evaluated only for Ag, while bioaccumulation detection is low and reproductive impairment is minimally represented. Acute mortality appears in about half of Ag-related responses and to a lesser extent for TiO_2_, reinforcing the emphasis on long-term physiological and developmental consequences over immediate lethality.

## Discussion

In principle, an LLM could extract every relevant parameter from an entire article. Full-texts in scientific articles often distribute key variables (e.g., concentration, exposure time, synthesis details) across distant sections: methods, results, supplementary notes, while also mixing original findings with cited or comparative data. However, automatically linking those dispersed pieces with high precision remains a challenge even for state-of-the-art LLMs when processing a single full-text article. Another key challenge we face with full text extraction is the difficulty of ensuring that a parameter we pull from the text genuinely reflects the original results of that article, rather than a comparative statement or a reference to another study. This issue is especially common in the Introduction and Discussion sections, where authors often compare their findings with previously published work. By working with abstracts at the sentence level, we ensure that each extracted parameter is directly traceable to its immediate textual context, avoiding attribution to external studies. We then perform a separate correlation step to connect related parameters. This modular design favors transparency, reproducibility, and error diagnosis, while maintaining a clear checkable trail. Finally, this approach that we are taking could also establish long-range text correlations between parameters without compromising reliability.

The common end points of the biological tests found in the corpus were MIC, MBC, LC_50_, IC_50_, EC_50_, and the percentage of inhibition (IP). Taking into account IC_50_ and EC_50_, we observed that these end points were used for both microorganisms and higher organisms, which can lead to confusion in their interpretation. In the context of nanotoxicology, IC_50_ (inhibitory concentration 50%) represents the concentration of a nanomaterial at which 50% of a measured biological activity (such as enzyme function, cell viability or microbial growth) is inhibited compared to a control. It is widely used in pharmacology, cell biology, and sometimes microbial assays when a graded response can be measured. In contrast, EC_50_ (effective concentration 50%) is the concentration at which 50% of the maximum effect is observed. This end point might refer more broadly to any quantifiable effect (such as mortality in an aquatic toxicity test), while IC_50_ is defined around “inhibition”, commonly of growth or a particular biological function. Both metrics measure a partial 50% response, but differ in terminology and context depending on whether to focus on “inhibition” (IC_50_) or a more general “effect” (EC_50_). IC_50_ and EC_50_ are widely used in pharmacology and ecotoxicology studies, where researchers measure half-maximal responses in eukaryotic cell cultures or aquatic test organisms such as *D. magna*, zebrafish, and others. In these contexts, an inhibitory or effective concentration 50% is a well-established measure of partial toxicity or reduced viability. In contrast, microbial research has conventionally relied on end points such as MIC and MBC, which focus on complete growth inhibition or bactericidal activity at the lowest possible concentration. However, in the corpus we found that some authors reported IC_50_ or EC_50_ values for bacteria (e.g., *E. coli*, *S. aureus*) when they measure a graded dose–response relationship, such as a 50% reduction in colony-forming units, optical density or metabolic activity. This overlap in the usage of end points in the corpus captured different degrees of toxicological or antimicrobial effects, making comparisons currently difficult. This is why we chose to use MIC and MBC for comparisons regarding microbial interactions with nanomaterials. Finally, LC_50_ (the lethal concentration that kills 50% of test organisms) was used primarily as an end point for higher organisms such as fish, aquatic invertebrates, or insect larvae. In those cases, mortality can be tracked directly as the end point, e.g., whether the organism is alive or dead after a set exposure period.

Taking into account the most available end points related to the interaction between nanomaterials and microorganisms (that is, minimum inhibitory concentration and minimum bactericidal concentration), we found significant differences in toxicity between Ag- and ZnO-based nanomaterials, which were the most studied in the corpus. Ag nanomaterials are more toxic than those with ZnO, regardless of the species considered. Ag-based nanomaterials, especially Ag nanoparticles, have antimicrobial effects because they release positively charged silver ions (Ag^+^) that bind to components containing sulfur and phosphorus in microbial cells, inactivating proteins, disrupting DNA, and ultimately hindering vital cellular processes^9,19^ Their size also enables strong interactions and penetration into cell membranes, which also causes structural damage and leakage of cellular components. In addition, silver nanoparticles can catalyze the formation of reactive oxygen species (e.g., hydroxyl radicals) which induce oxidative stress and damage lipids, proteins, and nucleic acids.

In contrast, ZnO nanomaterials display photo-oxidative and photocatalytic activity toward both chemical and biological targets. Their antimicrobial effects are influenced by UV illumination, size, concentration, morphology, lattice defects, and surface functionalization.^57^ The bactericidal and bacteriostatic mechanisms can be driven by reactive oxygen species (ROS), notably hydrogen peroxide (H_2_O_2_), hydroxyl radicals (·OH) and superoxide/peroxide species (*O*_2_^–^/*O*_2_^2–^). ROS-mediated cell wall injury, increased membrane permeability, nanoparticle internalization after loss of proton-motive force and the influx of toxic Zn^2+^ ions collectively compromise mitochondrial function, cause leakage of intracellular contents and trigger oxidative stress gene responses, ultimately suppressing microbial growth and leading to cell death.^58^

Furthermore, Ag-based nanomaterials were more toxic than those of TiO_2_, considering all microorganisms studied in the corpus (see Figure S8a in the Supporting Information). Photocatalytic excitation of TiO_2_ promotes electron–hole separation on the surface of the material, causing the formation of reactive oxygen species (ROS). These oxidants trigger lipid peroxidation cascades, protein carbonylation, and DNA strand breaks, ultimately leading to membrane leakage, genotoxicity, and cell death.^59^ TiO_2_ nanomaterials can also dissolve slowly, releasing Ti­(IV) ions that bind to phosphates and nucleotides, and their strong surface charge favors adsorption to cellular membranes, increasing local ROS flux and mechanical damage.^60^

Au-based nanomaterials, especially Au nanoparticles, also exhibit antimicrobial properties, but generally this property is reported to be weaker than Ag-based nanomaterials, mainly because it is considered that gold is chemically more inert and does not release ions in the same manner as silver. However, studies indicate that gold nanoparticles can disrupt bacterial cell membranes and produce instabilities in the microbial cell.^61,62^ Modifications such as functionalization of Au nanoparticles can significantly enhance their bactericidal potential. We observed here that mean values for end points related to antimicrobial effects of Au-based nanomaterials were close to those reported for nanomaterials with Ag. Through approaches like surface functionalization (e.g., grafting polymers, biomolecules, or small molecules), these nanoparticles can be anchored onto various substrates including carbon-based materials (graphene, nanotubes), oxides (e.g., TiO_2_, ZnO), or other metallic nanoparticles. This hybridization can be driven by electrostatic interactions, covalent bonding, or physical adsorption, resulting in structures that synergistically integrate the distinct properties of each component.^63,64^

The dominance of biological tests with *S. aureus* and *E. coli* in the corpus can bias the analysis with direct implications for environmental risk assessment. Although microbial assays are widely used in nanotoxicology, they primarily measure acute growth/viability end points in simplified media and therefore do not capture more complex processes such as bioaccumulation, trophic transfer, and developmental or reproductive toxicity. Consequently, species-sensitivity distributions and hazard thresholds derived from these data alone can underestimate risks to higher trophic levels and complex ecosystems. To address this, we analyzed the results separately by taxonomic group and highlight the need for targeted studies and chronic end points in standard ecotoxicological models to support cross-taxa extrapolation.

As the field of nanotechnology expanded, so did the need to assess the safety of engineered nanomaterials in a variety of model organisms to understand how different nanoparticles can affect living systems, from simple invertebrates to higher plants and vertebrates. Each model organism offers distinct advantages in terms of cost, experimental throughput, regulatory value, and relevance to human or environmental health. In addition, they reflect a spectrum of biological complexity, thereby ensuring a comprehensive approach to safety assessment. *D. magna* is a small freshwater crustacean (often called a water flea) widely recognized as a sentinel species for aquatic environments. Due to its filter feeding mechanism, the uptake of the nanoparticles is very likely, once the materials disrupt the gut cells barrier, they induce several toxic effects.^65^ For this freshwater crustacean, our LLM sentence analyzer evaluated acute toxicity (mortality and immobilization over 24 to 48 h) and chronic effects (reproductive output, time to first feces, and growth over a longer time scale). It also captures morphological or developmental changes—any reported anatomical malformations or sublethal stress—plus potential changes in biochemical markers of toxicity (e.g., catalase, superoxide dismutase, DNA damage). Behavioral end points, such as altered swimming or feeding, can be equally telling. If none of these adverse indicators appears or if the text explicitly mentions no observed effect (NOEL/NOAEL), the LLM model concluded that no toxic response was detected. In our results, more than 60% of the sentences that describe toxicity evaluations against *D. magna* indicated the existence of toxic effects for a variety of nanomaterial compositions.^66^

*D. rerio* (zebrafish) allows the evaluation of nanoparticle exposure from embryonic life stages to adult organisms, allowing the evaluation of various pathways of nanomaterial uptake and adverse developmental outcomes.^67^ For zebrafish, the sentence analyzer considered both short-term mortality or abnormal hatching and long-term effects on growth, reproduction, and organ development. It also checked for deformities (e.g., abnormalities in the skeletal or swimming bladder) and any mention of oxidative stress or genotoxicity (ROS production, DNA damage, micronuclei). Behavioral changes, such as altered swimming patterns or feeding behavior, are particularly relevant as sublethal indicators. For this species, similar results were observed compared to *D. magna*, where all the major core compositions of the nanomaterials led to considerable indications of toxicity.

The same was observed for *C. elegans*. Due to its short life cycle, well-mapped genome, and fully characterized nervous system, *C. elegans* offers a powerful platform to study the sublethal impacts of nanomaterials.^68^ In this case, the LLM analyzed acute and chronic toxicity. For short-term exposures, it looks at mortality or decreased viability over a day or two, while for long-term tests it considers changes in reproduction (e.g., smaller brood size), delayed development, and anatomical malformations. The analyzer also monitors oxidative stress indicators (e.g., ROS production, changes in antioxidant enzymes) and possible genotoxic effects such as DNA damage. Behavioral end points, such as reduced locomotion or feeding deficits, can also indicate sublethal toxicity in nematodes.

The knowledge on nanoparticle phytotoxicity is equally crucial to ensure ecological safety in the application of nanomaterials, especially considering their potential application in crop production. *A. thaliana* is a widely studied plant model with a fully encoded small genome as one of its main advantages. Furthermore, it presents an orthology with many mammalian genes responsible for DNA maintenance.^69^ Toxicity indications against *Arabidopsis* were less present, especially for core compositions such as Au, C and Fe_2_O_3_. To obtain this result, the LLM model analyzed impacts primarily on germination and plant growth, which are the main indices for the assessment of plant toxicity.^70^ Specific end points include reduced or delayed seed germination, diminished shoot or root elongation, and lower biomass production. Physiological and biochemical changes also play an important role, such as a decreased chlorophyll content, altered photosynthetic efficiency, and oxidative stress markers (e.g., increased ROS or lipid peroxidation). In addition, the sentence analyzer searched for evidence of leaf deformities, necrosis, chlorosis, or other morphological changes that signal a negative effect on plant health.

In toxicological terms, smaller nanoparticles are often more reactive and capable of crossing biological barriers, which can lead to a greater biological impact. This helps explain why some of the most toxic materials observed are among the smallest in size. Here, we observed more pronounced biological effects for smaller nanomaterials (≤50 nm), such as seen in the MIC and LC50 end points. However, the lack of a straightforward size-toxicity relationship also highlights the influence of other factors. A strong context dependence of toxicity typically overwhelms the simple size effect. Parameters such as surface chemistry, functionalization, ζ potential, agglomeration state, delivered dose, dissolution/ion release, protein corona formation, exposure medium (pH, ionic strength, organic matter) and duration of exposure must be considered. This aspect will require future stratification in the instantiation of a nanomaterial entity in the databases to achieve a complete description of this entity. To do this, there is a need to improve the relation-extraction capacity of the models, especially LLMs. Each nanomaterial property (e.g., size, charge, morphology, functional groups, synthesis route, etc.) must be considered. A future promising approach is the use of a well-refined ontology so that every property and relation is formally defined with canonical labels, synonyms, and unit rules. LLMs can understand this ontology either via few-shot prompting with a library of high-quality exemplars or a small instruction-fine-tune/RLHF (reinforcement learning from human feedback) pass that blends annotations with general tasks. It is important to increase the detail of toxicity against more complex organisms such as *D. magna*, *D. rerio*, *C. elegans* and *A. thaliana*, for which dozens of possible end points are analyzed (acute toxicity, chronic effects, behavioral changes, genotoxic effects, physiological and biochemical changes, and others).

A better description of the nanotechnological entity could be achieved by improving the extraction pipeline with a domain-specific ontology. Current standards such as Nanoparticle Ontology (NPO) and eNanoMapper can be used.^71,72^ In this way, each mention of a nanomaterial could be assigned to a unique identifier with rich synonyms, explicit measurement units, and a linked physicochemical profile (core composition, crystal phase, size, aspect ratio, surface area, zeta potential), exposure context (medium chemistry, ionic strength, presence of organic matter) and biological interface (species, toxicology end point, corona description, uptake pathway).^73^ By encoding the nanomaterials as an ontology-grounded node enriched with well-defined physicochemical, exposure, and biological interface attributes, we can establish structured knowledge graphs with observed toxic outcomes and experimental contexts. This ontology-centric graph approach converts heterogeneous textual evidence into an interpretable, machine-readable substrate, allowing data-efficient and mechanistically transparent prediction of nanomaterial toxicity across diverse species and end points.^74^

The primary goal for ontology use is the stratification of extracted data rather than the imputation. Ontologies provide controlled identifiers for (i) nanomaterial descriptors (core composition, crystal phase, morphology, porosity, surface modifiers, and functionalization) and (ii) test context (test species, medium/pH/ionic strength/serum, exposure regime, and end point definition). For example, obtain “TiO_2_ NP” and normalize it to a single core class (TiO_2_), phase (anatase/rutile/mixed) and surface (e.g., PEG, PVP, amine), while simultaneously normalizing the test conditions and end points (e.g., IC_50_/EC_50_/LC_50_). This ontology-backed normalization enables species- and condition-aware strata for analysis and modeling, which we have found to be essential to reduce confounding and improve predictive accuracy. This approach is very promising when using full-length articles instead of abstracts.

However, it must be considered that there is an issue with the completeness of the nanomaterial characterization reported in the primary literature, both at the abstract and full-length article scale. Taking into account the physicochemical aspects, a proper characterization of the nanomaterial entity would involve properties such as size, composition, morphology, crystallographic phase, surface area, surface charge (ζ potential), dissolution rate, colloidal stability, corona characteristics (if present) and surface functional groups.^75^ This incompleteness makes it difficult to reproduce experiments, compare nominally similar materials in studies, and mechanistically interpret biological results. Meta-analyses and machine learning efforts are also hindered.^76^ This problem persists despite community guidelines because detailed characterization may require instrumentation beyond the reach of many laboratories. As a result, current NLP approaches with LLM-based extraction pipelines must not only identify the properties that are reported, but also flag those that are absent in order to fill the gaps with uncertainty annotations, future experiments, and interface with external databases or predictive tools.^77,78^

## Conclusions

In this article, we demonstrate that LLM-based NLP pipelines can systematically mine the nanotoxicology literature at a scale and resolution unattainable with manual curation alone. By coupling end point-specific extraction prompts with a second-stage validator prompt, we converted approximately 1 million sentences into a structured knowledge base spanning 13 features related to nanotoxicology. Multiple statistically analyzed quantitative end points converge on the same conclusions about the interactions of nanomaterials and microorganisms. Ag-based nanomaterials are the most potent antimicrobial agents in the corpus. They have lower values of minimum inhibitory concentration (MIC) and minimum bactericidal concentration (MBC) compared to other widely studied nanomaterials such as ZnO, TiO_2_ and Au. The same conclusion was achieved when other end points were used (e.g., microbial log-reduction and biofilm killing efficiency). This trend continued even after filtering the MIC values for individual pathogens such as *E. coli* and *S. aureus*.

The majority of nanomaterials had sizes between 1 and 100 nm, with morphologies that include nanoparticles, nanotubes, dots, nanorods, nanosheets, nanofibers, nanowires and nanospheres. Despite the variety of morphologies, nanoparticles were present in more than 80% of the articles. On the nanomaterials size effects, although nanomaterials with sizes smaller than 50 nm often yielded the lowest MIC and LC_50_ values, toxicity within a single size class spanned orders of magnitude, underscoring the role of other nanomaterial features like surface chemistry, coatings, and colloidal behavior. On the interactions with superior organisms, the majority of articles report impairment in the reproduction of *C. elegans* and *D. magna* induced by Ag and TiO_2_ nanomaterials. These same nanomaterials can cause developmental alterations and morphological abnormalities in *D. rerio*, as indicated in most articles dealing with these interactions.

Finally, our NLP/LLM-driven pipeline demonstrates that valuable toxicological information can be extracted, including nanomaterial descriptors such as size, ζ potential and surface area, and biological end points such as MIC, MBC and percentage of biofilm destruction. However, the next step toward predictive models will come from conjugating this extraction framework to a domain-specific ontology that assigns every nanomaterial a unique, richly annotated identity. Such an ontology-centric extraction would not only unify heterogeneous evidence but also allow mechanistically transparent toxicity forecasts, especially when extended to full-text corpora. However, this approach will require systematic attention to a persistent obstacle, that is, incomplete or inconsistently reported characterization data. Future NLP tools must therefore flag missing attributes, attach uncertainty annotations, and, where possible, impute values from external databases or follow-up experiments, ensuring that the resulting knowledge base accurately captures the hazards of engineered nanomaterials.

## Supplementary Material



## Notes

Information available in the Supporting Information includes details (i) about the database search; (ii) LSA and LDA algorithms; (iii) settings to run aRIX routine searches and consolidate extracted dataframes; (iv) extraction accuracy test; (v) results of LSA and LDA topic modeling; (vi) term frequencies results; (vii) box-plots and Welch statistical tests results; (viii) scatter plots and (ix) tables with the extracted values (PDF)
