# Supplementary material for: Large-Scale Meta-Analysis of Nanomaterials Toxicity Based on Natural Language Processing of Scientific Articles
Source: ACS Appl Nano Mater. 2025 Dec 23;9(1):28–44. doi: 10.1021/acsanm.5c05119 (PMC12797190; doi:10.1021/acsanm.5c05119)
Supplement: Supplementary file 1 [file an5c05119_si_001.pdf]

Supporting Information
Large-Scale Meta-Analysis of Nanomaterials Toxicity Based on Natural Language Processing of Scientific Articles

Amauri J. Paula,[*,†] Romana Petry,[*,‡] James M. Almeida,[¶] André A. Caetano,[¶] José Sales,[¶] Odair P. Ferreira,[§] Diego S.T. Martinez,[‡] Henry J. Kobs,[‖] and Andreia F. Faria[‖]

†*Solid-Biological Interface Group (SolBIN), Aeronautics Institute of Technology - ITA, São José dos Campos, 12228-900, SP, Brazil*

‡*Brazilian Nanotechnology National Laboratory (LNNano), Brazilian Center for Research in Energy and Materials (CNPEM), Campinas, 13083-100, SP, Brazil*

¶*Ilum School of Science (Ilum), Brazilian Center for Research in Energy and Materials (CNPEM), Campinas, 13087-548, SP, Brazil*

§*Laboratory of Advanced Functional Materials (LaMFA), Department of Chemistry, State University of Londrina (UEL), Londrina, 86055-900, SP, Brazil*

‖*Engineering School of Sustainable Infrastructure & Environment, Department of Environmental Engineering Sciences, University of Florida, Gainesville, 32611-6580, FL, United States*

E-mail: amaurijp@ita.br; romana.petry@lnnano.cnpem.br

Contents

1 Methods **S-3**

1.1 Database search S-3

1.2 aRIX settings S-4

1.3 Fields “semantic_entry” and “parameter_to_extract” S-5

1.4 LSA and LDA algorithms S-5

1.5 Extraction accuracy test S-6

2 Results **S-11**

2.1 Topic modeling S-11

2.2 Nanomaterials properties S-34

2.3 Interactions with microorganisms S-37

2.4 Interactions with superior organisms S-43

2.5 Extracted values - Full tabular data S-45

1 Methods

1.1 Database search

We performed a targeted search on the Clarivate Web of Science (WOS) database using combinations of terms and Boolean operators. The main concept of the search was to access articles that indicated in the title, keywords, or abstract the presence of nanomaterials and the existence of possible nanotoxicity assays performed with well-known biological models. The search terms are as follows:

TS(Topic Search)=
(“dendrimer” or “dendrimers” or “fullerene” or “fullerenes” or “graphene” or “graphenes” or “*dot” or “*dots” or “nanocapsule” or “nanocapsules” or “nanocube” or “nanocubes” or “nanodisk” or “nanodisks” or “nanofiber” or “nanofibers” or “nanohelix” or “nanohelixes” or “nanoparticle” or “nanoparticles” or “nanoribbon” or “nanoribbons” or “nanorod” or “nanorods” or “nanosheet” or “nanosheets” or “nanosphere” or “nanospheres” or “nanostar” or “nanostars” or “nanostructure” or “nanostructures” or “nanotube” or “nanotubes” or “nanowire” or “nanowires”)
AND
(“African clawed frog” or “Arabidopsis” or “Arabidopsis thaliana” or
“Arthrobacter globiformis” or “B. subtilis” or “Bacillus subtilis” or “Brassica juncea” or “C. elegans” or “C. vulgaris” or “Caenorhabditis elegans” or “Chlorella vulgaris” or “Clover species” or “D. carinata” or “D. galeata” or “D. lumholtzi” or “D. magna” or “D. pulex” or “D. rerio” or “D. similis” or “Danio rerio” or “Daphnia carinata” or “Daphnia galeata” or “Daphnia lumholtzi” or “Daphnia magna” or “Daphnia pulex” or “Daphnia similis” or “Drosophila” or “Drosophila melanogaster” or “Duckweed” or “E. coli” or “Earthworms” or “Eisenia fetida” or “Escherichia coli” or “Fathead minnow” or “Folsomia candida” or “Freshwater snails” or “Green algae” or “Green hydra” or “Hydra viridissima” or “Lemna minor” or “Lolium perenne” or “Lymnaea stagnalis” or “Mustard plant” or “Oncorhynchus mykiss” or “Pimephales promelas” or “Rainbow trout” or “Raphidocelis subcapitata” or “Ryegrass” or “S. aureus” or “S. cerevisiae” or “Saccharomyces cerevisiae” or “Selenastrum capricornutum” or “Springtails” or “Staphylococcus aureus” or “Trifolium pratense” or “Xenopus laevis”

or "Zebrafish" or "african clawed frog" or "clover species" or "drosophila" or "duckweed" or "earthworms" or "fathead minnow" or "freshwater snails" or "green algae" or "green hydra" or "mustard plant" or "rainbow trout" or "ryegrass" or "springtails" or "zebrafish")

1.2 aRIX settings

To make our work fully auditable and reusable, we have published a public repository on GitHub (github.com/amaurijp/nanotox_db), which contains (i) the complete list of DOIs for each article in our corpus and (ii) all the configurations needed to reproduce the search-extract (SE) routines and the subsequent consolidation steps used in the manuscript. See the details below:

- `abstracts_filename_id_1.csv` lists all articles included in our study with their DOI identifiers.
- `SE_inputs.csv` specifies the SE routines that select sentences containing relevant information to be extracted (targets, patterns, and normalization rules) and the extracted parameters performed via regex patterns or LLMs prompts.
- Each configured SE routine writes a CSV file with the values of the extracted parameters (numeric or categorical) and their sentence index. These routine-level CSVs are included in the repository.
- Routine-level CSVs are harmonized and merged into analysis-ready tables; see the files:
  - `DFs_to_consolidate*.csv`
- The final consolidated dataset used to generate the figures in the paper is:
  - `abstracts_nanotox_db_all_1.csv`
  - `abstracts_nanotox_db_superior_organisms_1.csv`

With the manifest of DOIs and SE/consolidation routines, readers and reviewers can (a) trace any data point back to its source sentence, (b) rerun the extraction and consolidation steps, and (c) apply alternative normalization or filtering choices using the same inputs. Finally, the code used in the manuscript is on github.com/amaurijp/aRIX (along with the documentation).

1.3 Fields “semantic_entry” and “parameter_to_extract”

For this article, we set specific categorical parameters (see the list on Github). Each of these categories has a list of ngrams that are searched and extracted if any of them are found in sentences or abstracts. The term lists were obtained by finding cosine similarities among token word vectors (generated by the Word2Vec approach). In the case of species-related categories (e.g., “species_genus_all_abbr”, “species_genus_all_full” or “species_toxicological_models”), each 2gram found through the analysis of word vectors was checked for correctness in the National Center for Biotechnology Information (NCBI) databases. Categories and term lists are organized in the file “.../Inputs/ner_rules.json”.

1.4 LSA and LDA algorithms

LSA generates the document-topic and token-topic matrices through the singular value decomposition (SVD) of the TFIDF matrix obtained in the previous step. To obtain these matrices, the process begins with the construction of a token-document matrix, where rows represent tokens and columns represent documents. Each entry in the matrix contains the TF-IDF value that reflects the importance of each token in the document. Singular Value Decomposition (SVD) is then applied to factorize the token-document matrix into three matrices: $\mathbf{U}_{M,M}$ (representing tokens vs. topics), $\sigma_{M,Y}$ (containing singular values), and $\mathbf{V}_{Y,Y}$ (representing topics vs. documents), where Y can be the total number of sentences (N) or the total number of abstracts (R). The token-topic matrix is the truncated version of $\mathbf{U}_{M,M}$ (i.e., $\mathbf{U}_{M,d}$), where only the top d singular values are kept to reduce the dimensionality, specifically those with larger values (we used $d = 100$ in this article). On the other hand, the document-topic matrix is obtained by multiplying the document-token matrix ($TFIDF_{Y,M}$) by matrix $\mathbf{U}_{M,d}$.

LDA is a probabilistic model for topic modeling that represents documents as mixtures of topics, where each topic is a distribution over tokens. Using a collapsed Gibbs Sampling algorithm for LDA, we started by iteratively sampling topic assignments for each token in each sentence and abstract of the corpus. Initially, each token in a document (sentence or abstract) is randomly assigned a topic number (here it is used between 1 and 100), and thus a document-token-topic matrix is created. In

addition, two count matrices are created to track the number of occurrences of each token in each topic (token-topic-count matrix) and the number of occurrences of each topic in each document (topic-document-count matrix). For each token, its topic assignment is temporarily removed, and a new topic is sampled based on the likelihood of the topic appearing in the document and the token's likelihood in the topic. After sampling, the counts are updated, and this process is repeated over 200 iterations until convergence. The token-topic vectors used to generate the topic vector T_i are the lines of the token-topic count matrix created during Gibbs sampling. The document-topic matrix is the matrix in which the topics are assigned during Gibbs sampling. The document-topic matrix reflects the probability of topics in documents, and the topic-token matrix shows the probability of tokens in topics.

1.5 Extraction accuracy test

The accuracy of each search-extraction (SE) routine, as well as the consolidation routine, was assessed in three steps:

1. All search-extract (SE) routines (see all here) that used LLM in the final extraction step was first checked. For each parameter extracted in each search/extract routine, we have a JSON file with a set of annotated sentences (with the respective DOI) that were used to check the accuracy per routine (see here). The consolidated result is shown in Table S1. A total of 1475 sentences was used in this first accuracy test. This test did not apply to the simpler routines that used just regex patterns for extraction (i.e., extracting the word "carbon" from a sentence).

2. After extraction with different SE routines was performed, we performed the consolidation routine for the parameters in a single column of the database (see all here and here). For example, both SE routines "microbe_killing_mic_01" and "microbe_killing_mic_02" were used to extract values of the minimum inhibitory concentration against microbes, although with different configurations of the aRIX program. Thus, these two SE routines are consolidated through a consolidation routine for the parameter "microbe_killing_mic".

3. With the consolidated parameters, we performed another accuracy test for the LLM prompt

that was used to determine whether the parameters extracted during the SE routines were correct and whether they were properly correlated. The annotated texts (with the respective DOI) and the prompts used in this step are available in JSON files here. A total of 275 sentences was used in the evaluation. Accuracy results for the consolidated dataframes are shown in Table S2. The final minimum accuracy value after this filter step was 94% considering all the parameters extracted.

Table S 1: Accuracy test performed on the each search/extraction routine that made use of LLMs.

SE routine name	Accuracy
2dmaterials_01	1.0
2dmaterials_02	1.0
biofilms_killing_perc_01	1.0
biofilms_killing_perc_02	0.96
metallic_nano_01	0.88
metallic_nano_02	0.76
microbe_killing_log_01	1.0
microbe_killing_log_02	1.0
microbe_killing_mbc_01	1.0
microbe_killing_mbc_02	0.96
microbe_killing_mic_01	0.96
microbe_killing_mic_02	1.0
nps_size_01	0.88
oxidesmaterials_01	0.76
qdots_01	0.96
species_athaliana_02	1.0
species_athaliana_04	0.92
species_athaliana_06	0.96
species_athaliana_08	1.0

SE routine name	Accuracy
species_athaliana_10	0.96
species_athaliana_12	1.0
species_athaliana_14	0.96
species_athaliana_16	1.0
species_athaliana_18	1.0
species_athaliana_20	1.0
species_celegans_02	1.0
species_celegans_04	1.0
species_celegans_06	0.92
species_celegans_08	1.0
species_celegans_10	1.0
species_celegans_12	1.0
species_celegans_14	0.92
species_celegans_16	1.0
species_celegans_18	1.0
species_celegans_20	1.0
species_dmagna_02	0.8
species_dmagna_04	0.76
species_dmagna_06	0.84
species_dmagna_08	0.92
species_dmagna_10	0.8
species_dmagna_12	1.0
species_dmagna_14	0.84
species_dmagna_16	0.64
species_dmagna_18	1.0
species_dmagna_20	0.76

SE routine name	Accuracy
species_drerio_02	0.72
species_drerio_04	0.64
species_drerio_06	0.92
species_drerio_08	1.0
species_drerio_10	0.64
species_drerio_12	0.92
species_drerio_14	0.52
species_drerio_16	0.67
species_drerio_18	0.68
species_drerio_20	0.88
surface_area_01	1.0
toxic_lc50_01	1.0
toxic_lc50_02	1.0
zetapot_01	0.88

Table S 2: Accuracy test performed on correlation that made use of LLMs.

Consolidation routine name	Parameters with correlation checked	Accuracy
biofilm_killing_perc	nanomaterial_composition<br>biological_species<br>biofilm_killing_perc	1.0
biological_species	nanomaterial_composition<br>biological_species	0.96
microbe_killing_mbc	nanomaterial_composition<br>biological_species<br>microbe_killing_mbc	0.96
microbe_killing_mic	nanomaterial_composition<br>biological_species<br>microbe_killing_mic	0.96
nanomaterial_morphology	nanomaterial_composition<br>nanomaterial_morphology	1.0
nanomaterial_size	nanomaterial_composition<br>nanomaterial_morphology<br>nanomaterial_size	0.94
nanomaterial_surface_area	nanomaterial_composition<br>nanomaterial_morphology<br>nanomaterial_surface_area	1.0
nanomaterial_zeta_potential	nanomaterial_composition<br>nanomaterial_morphology<br>nanomaterial_zeta_potential	0.94
toxicity_yes_no	nanomaterial_composition<br>biological_species<br>toxicity_endpoints<br>toxicity_yes_no	0.96

2 Results

2.1 Topic modeling

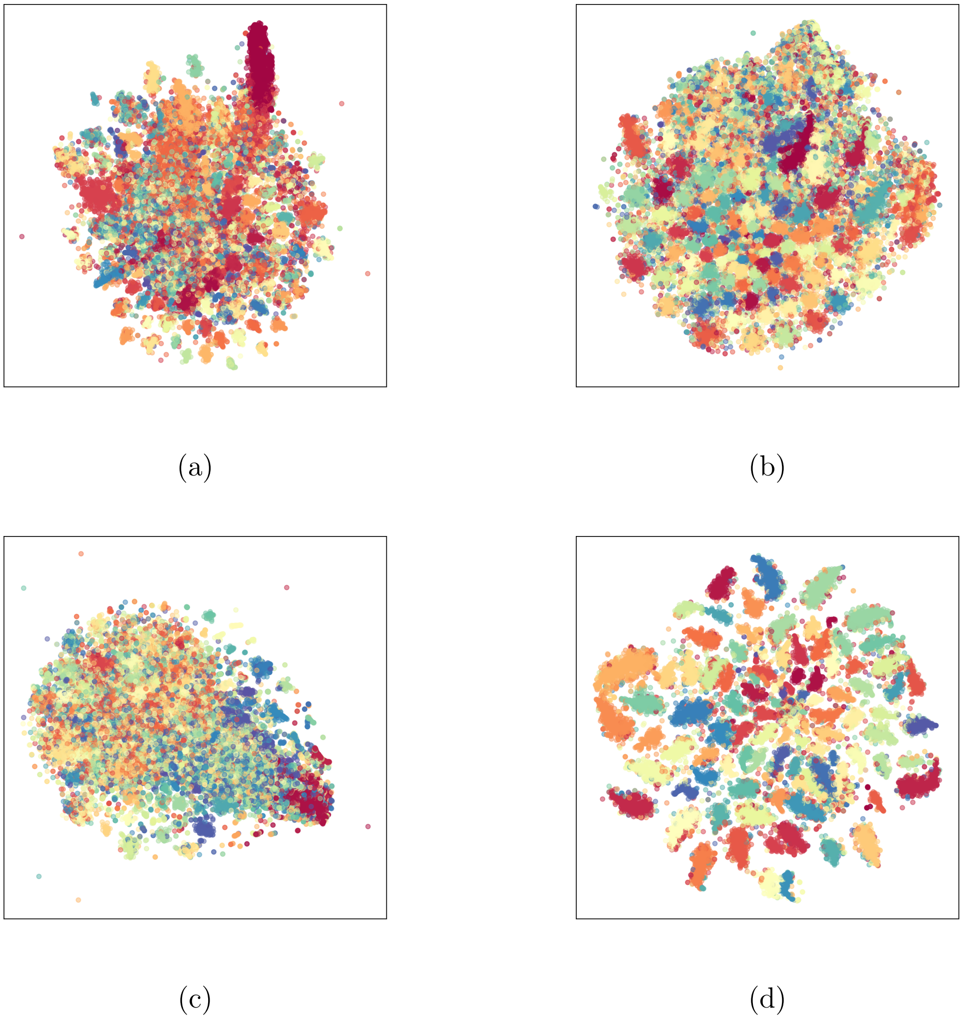

Figure S 1: Representations of the token-topic matrices in a reduced dimension (2D) obtained via t-distributed stochastic neighbor embedding (t-SNE). Topics were modeled at the abstract level with (S1a) LSA and (S1b) LDA methods, and at the sentence level with (S1c) LSA and (S1d) LDA. The original dimension of the token-topic matrices were of 23997 (number of unique tokens) x 100 (number of topics). Tokens in the representation are colored based on the major topic they are related to. The topic modeling was performed at the sentence level, i.e., sentences were considered to be the document in the document-token matrices used during LSA and LDA modeling.

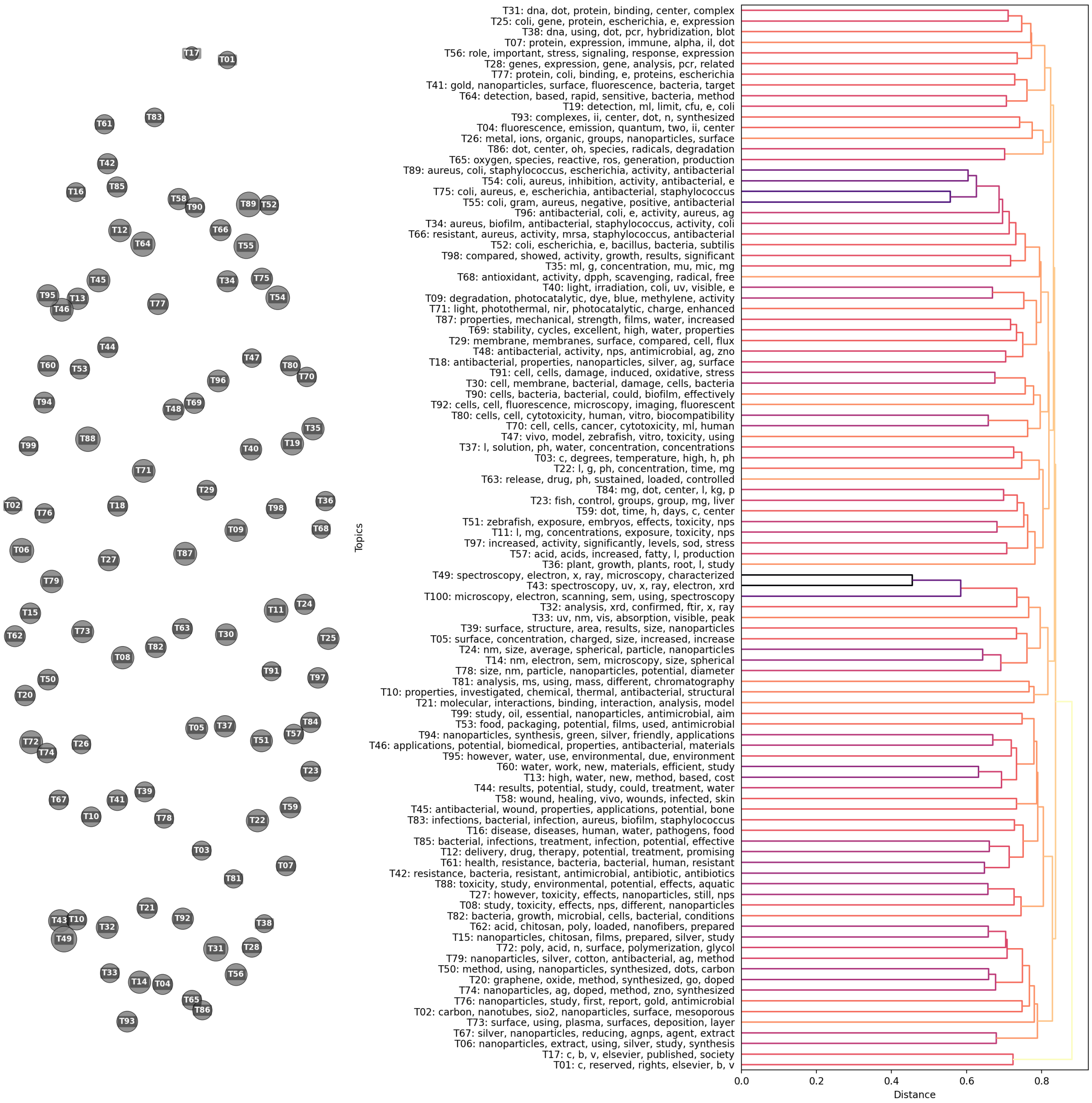

Figure S 2: (Left) A two-dimensional representation of the topic distribution and their relative distances, illustrating how topics are spread and clustered in the chosen dimensionality reduction space. The size of the gray circles represents the importance of the topic across the entire corpus. (Right) A dendrogram depicting the hierarchical relationships among the identified topics, highlighting how topics group together.

Table S 3: Relation between the topics identified via LSA at the abstract level and the 15 most frequent tokens associated to each topic

Topic number	tokens with most occurrences
1	coli, study, aureus, escherichia, using, activity, results, staphylococcus, showed, nanoparticles, used, potential, antibacterial, e, also
2	zno, nps, ag, tio2, cuo, cu, silver, au, aureus, staphylococcus, nano, np, ceo2, doped, coli
3	agnps, zno, nps, results, coli, based, c, escherichia, high, also, e, elsevier, study, reserved, rights
4	ag, tio2, nps, agnps, aureus, coli, staphylococcus, escherichia, cu, cuo, au, go, antibacterial, bacteria, scanning
5	go, ag, agnps, zno, cs, study, hap, rgo, pva, used, using, results, c, also, membrane
6	ag, study, tio2, results, however, nanoparticles, films, used, effects, potential, also, cs, cotton, c, pva
7	tio2, zno, agnps, go, films, nano, nanoparticles, silver, doped, n, aureus, staphylococcus, nm, potential, anatase
8	ag, zno, cds, electron, x, agnps, ray, microscopy, scanning, diffraction, qds, fourier, infrared, spectroscopy, transform
9	cds, qds, nps, study, go, results, cdte, c, zns, cdse, cd, n, showed, however, elsevier
10	cu, cuo, nps, center, dot, cds, cs, electron, films, pva, microscopy, x, agnps, scanning, ray
11	center, dot, ag, qds, aureus, coli, nanoparticles, staphylococcus, complexes, oh, cl, n, zno, escherichia, h2o
12	cu, cuo, qds, nanoparticles, aureus, ag, ii, complexes, staphylococcus, potential, agnps, study, cotton, dna, silver
13	qds, cs, pva, cdte, cu, zns, cd, coli, cdse, escherichia, films, nanofibers, pla, bacteria, qd
14	qds, nps, cuo, cdte, cotton, zns, cdse, fabrics, dna, fabric, au, electron, scanning, x, ray
15	c, cu, elsevier, reserved, rights, qds, b, v, escherichia, ag, tio2, coli, rgo, cdte, zno
16	cuo, rgo, ag, c, nanoparticles, escherichia, np, zno, elsevier, staphylococcus, reserved, rights, films, b, coli
17	films, cd, nanoparticles, pva, silver, cotton, nanofibers, chitosan, se, pla, coli, fabrics, escherichia, nps, ps
18	cotton, fabrics, fabric, cs, silver, c, cd, elsevier, coated, antibacterial, staphylococcus, polyester, reserved, rights, fibers
19	aureus, staphylococcus, films, dna, c, rgo, elsevier, reserved, rights, antibacterial, b, v, mgo, cs, aunps
20	rgo, films, cotton, escherichia, coli, fabric, fabrics, pva, nps, se, hydrogel, nanocomposite, using, e, film
21	hap, rgo, cd, nanoparticles, zn, nps, se, silver, agnps, complexes, ii, ceo2, pcl, beta, staphylococcus
22	hap, aunps, films, cotton, dna, fabrics, au, fabric, escherichia, pva, detection, coli, sers, cuo, pla
23	cd, aunps, au, dna, e, beta, nanofibers, se, complexes, cuo, pva, coli, ii, zn, ha
24	au, bc, nanoparticles, pva, mgo, fe3o4, nanofibers, silver, coli, mwcnts, ha, ps, agnps, cotton, pani
25	mgo, bc, cd, aunps, nzvi, soil, beta, zn, ncs, using, nisin, np, agnp, results, rgo
26	au, cd, films, se, study, staphylococcus, nzvi, fe3o4, results, using, cotton, aureus, ncs, senps, mos2
27	bc, se, nzvi, senps, dna, fe3o4, hap, selenium, cs, mmt, composites, agnp, selenite, activity, cqds
28	se, mgo, au, senps, nanoparticles, ha, e, silver, selenium, nano, ncs, cuo, coli, dna, np
29	pva, nanofibers, se, staphylococcus, aureus, hydrogel, pda, dna, study, hydrogels, membranes, ha, pcl, senps, membrane

Topic number	tokens with most occurrences
30	nzvi, aunps, pva, soil, nanofibers, ha, se, vi, fe, hydrogels, using, u, qds, earthworms, silver
31	dna, nzvi, coli, e, mgo, study, films, complexes, escherichia, soil, ii, hydrogel, showed, pva, detection
32	ha, ceo2, dna, films, g, c3n4, pu, study, mos2, coatings, coating, bone, ta, ce, mu
33	ps, mps, ha, nzvi, np, transgenerational, using, results, fe3o4, microplastics, toxicity, pda, activity, nanoplastics, study
34	mwcnts, fe3o4, e, cnts, pda, mwcnt, activity, mir, f, toxicity, size, nm, nps, sers, o157
35	ceo2, mwcnts, ps, mps, dna, np, hydrogel, au, pva, c3n4, results, used, using, senps, g
36	fe3o4, ceo2, e, pda, sio2, coli, ps, magnetic, pla, se, o157, mnps, mps, h7, mgo
37	fe3o4, cqds, nanoparticles, aunps, pva, escherichia, dna, silver, mos2, pda, mwcnts, hydrogel, nps, study, hydrogels
38	pla, nanofibers, dna, nanoparticles, pcl, mwcnts, cqds, results, nzvi, se, silver, escherichia, aunps, mos2, fibers
39	cqds, e, o157, h7, activity, cd, g, pva, sers, detection, c3n4, antibacterial, se, showed, hap
40	ef, tu, cqds, trna, pla, ts, gtp, se, study, c3n4, nanofibers, g, coli, e, gdp
41	cqds, complexes, ii, senps, nanofibers, pla, n, zn, ni, c3n4, g, fe, agnp, pd, l
42	senps, cqds, pva, ha, ef, ceo2, mwcnts, silver, cd, tu, results, e, o157, trna, electron
43	senps, hydrogel, cqds, wound, hydrogels, g, silver, c3n4, using, dna, pda, healing, mu, ml, membranes
44	mos2, c3n4, senps, g, mu, l, pva, ml, nanofibers, silver, nio, coli, pani, e, showed
45	mos2, using, zn, pda, senps, pla, method, antibacterial, surface, au, cqds, ii, fe, complexes, zif
46	pla, senps, hydrogel, study, ii, silver, complexes, pda, hydrogels, fe, nanoparticles, o157, h7, zif, e
47	using, pla, pva, fe3o4, dna, c3n4, used, nanoparticles, zif, method, based, g, surface, e, ncs
48	pla, mos2, cunps, hydrogels, results, hydrogel, ncs, fe3o4, pva, nio, l, g, agnp, wound, pani
49	membranes, membrane, cunps, pes, pva, study, pani, escherichia, pvdf, coli, cell, pda, cells, silver, cu2o
50	ncs, mos2, membranes, membrane, pani, nio, nanoparticles, e, wound, pes, bacteria, agnp, activity, pcl, used
51	zn, results, ncs, zif, pda, nio, membranes, membrane, soil, silver, showed, activity, bacteria, pes, ef
52	ncs, cunps, zif, pda, silver, study, using, cur, pani, o157, h7, mos2, znonps, positive, pd
53	zn, nio, study, using, soil, ncs, cu2o, nanofibers, silver, chitosan, np, hydrogel, pani, senps, antibacterial
54	nio, pda, cur, mos2, ni, cdo, nanofibers, curcumin, nanocomposites, o157, h7, au, complexes, np, agnp
55	zif, nio, using, hydrogel, pcl, chitosan, membranes, used, hydrogels, o157, h7, bacteria, vi, sa, mos2
56	using, fe, results, cunps, silver, pd, c3n4, membranes, nio, pcl, ii, ni, e, fe3o4, iii
57	silver, np, agnp, pd, mnps, nisin, fe, results, ps, pani, snps, showed, nio, study, pt
58	agnp, c3n4, o157, cur, snps, h7, znonps, using, chitosan, curcumin, cu2o, also, film, results, cells
59	agnp, nanofibers, chitosan, mps, fe, showed, zif, n, pda, cunps, hydrogel, results, ncs, using, pani
60	pani, np, cur, nanofibers, pd, curcumin, o157, h7, activity, high, surface, hydrogel, hydrogels, pt, used
61	chitosan, nisin, using, np, sers, n, gqds, escherichia, complexes, l, detection, ncs, mg, results, mu
62	o157, h7, agnp, np, aureus, pcl, ps, e, complexes, staphylococcus, cunps, membranes, nanoparticles, study, wound

Topic number	tokens with most occurrences
63	snps, chitosan, pcl, mps, pd, l, o157, h7, pda, mg, aureus, staphylococcus, mnps, using, hydrogels
64	nisin, pd, mps, fe, pcl, agnp, mnps, zn, pani, nanoparticles, also, aureus, zif, cunps, staphylococcus
65	pani, chitosan, gqds, snps, n, bacteria, cunps, fe, cu2o, nanoparticles, c3n4, pda, pcl, cnts, results
66	gqds, n, pani, znonps, mps, silver, cu2o, complexes, l, pcl, mnps, study, mg, cnts, surface
67	cur, curcumin, pcl, chitosan, agnp, sers, peg, pani, study, pva, using, film, activity, l, scaffolds
68	cur, snps, curcumin, gqds, cu2o, membranes, pd, fe, zif, e, membrane, nanofibers, study, ncs, pes
69	snps, sers, vi, pani, using, silver, u, cr, eps, agnp, hydrogels, cur, activity, complexes, nisin
70	cu2o, pd, gqds, using, agnp, pcl, pt, ps, nanoparticles, sers, sio2, vi, copper, escherichia, potential
71	pd, bacteria, activity, snps, pani, sers, negative, agnp, positive, study, nanoparticles, complexes, nanofibers, cunps, wound
72	fe, gqds, l, ps, sers, used, beta, mg, ca, escherichia, ta, n, detection, nfs, using
73	znonps, results, l, cnts, fe, study, mg, cnt, ca, bacteria, e, ta, nanoparticles, hydrogels, ps
74	activity, gqds, pcl, znonps, c3n4, vi, fe, n, cd, chitosan, cr, u, pda, np, also
75	vi, u, cr, beta, gqds, results, l, mnps, zn, alpha, fe2o3, e, adsorption, ca, chitosan
76	mnps, hydrogels, c3n4, ca, cnts, ps, ta, cnt, activity, also, peg, bacterial, n, antibacterial, results
77	hydrogels, znonps, showed, sers, beta, pd, gqds, nisin, cnts, potential, nm, sio2, zn, cur, alpha
78	cu2o, activity, mnps, znonps, ps, l, hydrogels, chitosan, zn, ii, snps, eps, agnp, nisin, nano
79	used, n, cnts, cu2o, ps, cnt, sers, vi, mnps, potential, u, cr, cd, ta, aureus
80	used, ca, hydrogels, np, mps, sers, ta, cu2o, msns, pei, fibers, pd, eps, antibacterial, method
81	cnts, showed, cnt, hydrogels, np, gqds, bacteria, also, ni, gnps, mps, pei, vi, cd, film
82	sers, mnps, used, gqds, np, nanoparticles, pda, soil, e, cnts, curcumin, beta, potential, study, eps
83	sio2, used, nano, hydrogels, nfs, bacteria, results, pani, silica, copper, antibacterial, curcumin, negative, shell, fe
84	cnts, activity, also, fe2o3, beta, sio2, cnt, cu2o, alpha, np, used, eps, nfs, mps, silver
85	n, also, showed, sio2, mnps, np, e, hydrogels, nanoparticles, using, l, fe, bacteria, membranes, cu2o
86	ta, showed, soil, mnps, sa, znonps, nfs, used, fe2o3, sio2, nanoparticles, snps, np, bacterial, pcl
87	ca, cnts, used, n, fe2o3, activity, soil, showed, np, nanoparticles, o157, h7, znonps, cur, cnt
88	potential, l, bacteria, showed, fibers, fe2o3, mg, cuonps, wo3, sno2, curcumin, hydrogel, e, cells, silver
89	ca, also, potential, sio2, mnps, np, znonps, sers, showed, nanoparticles, snps, wound, l, chitosan, c3n4
90	soil, also, mg, l, kg, earthworms, used, antibacterial, ps, hydrogels, membranes, nm, beta, size, complexes
91	fibers, ca, e, sio2, soil, sers, bacterial, aureus, cells, wound, hydrogel, nano, beta, also, cell
92	eps, fibers, nfs, also, ti, sno2, film, cur, hydrogels, zein, n, mnps, wound, np, ch
93	curcumin, beta, nfs, sno2, antibacterial, cnts, znonps, n, hydrogel, np, mnps, g, fibers, ti, activity
94	nfs, sno2, mir, properties, cur, gnps, ti, surface, hydrogels, wound, cu2o, sers, pei, ca, chitosan
95	fibers, pei, mir, wound, hydrogels, curcumin, c3n4, properties, l, healing, surface, plga, potential, n, high
96	eps, copper, pei, curcumin, wo3, l, tc, nfs, cells, sno2, cell, membrane, pcl, showed, znonps
97	eps, fe2o3, soil, curcumin, ca, wound, gnps, nfs, ta, showed, hydrogels, alpha, high, g, sno2

Topic number	tokens with most occurrences
98	mir, eps, pei, potential, beta, film, hydrogel, sio2, ca, showed, pcl, bacteria, nanopolystyrene, toxicity, cells
99	mir, fe2o3, curcumin, ti, also, copper, hydrogel, nanopolystyrene, nfs, toxicity, mirnas, alpha, nano, high, cmc
100	copper, gnps, sno2, potential, peg, plga, pei, activity, fibers, znonps, wound, nc, g, soil, e

Table S 4: Relation between the topics identified via LSA at the sentence level and the 15 most frequent tokens associated to each topic

Topic number	tokens with most occurrences
1	reserved, rights, ltd, elsevier, c, inc, v, b, published, ireland, authors, science, author, materialia, acta
2	graphics, activity, antibacterial, nanoparticles, coli, bacteria, showed, e, bacterial, synthesized, aureus, take, para, surface, steps
3	coli, antibacterial, aureus, activity, nanoparticles, elsevier, escherichia, v, b, e, c, staphylococcus, bacteria, using, study
4	elsevier, v, b, c, published, ltd, authors, author, inc, gmbh, science, sas, masson, copyright, university
5	polym, sci, part, u, appl, periodicals, wiley, j, inc, society, b, industry, protein, colloid, interface
6	nanoparticles, using, study, silver, nps, electron, x, microscopy, ray, scanning, diffraction, synthesized, spectroscopy, characterized, xrd
7	sci, u, appl, part, coli, aureus, society, escherichia, staphylococcus, industry, e, protein, colloid, interface, activity
8	appl, biomater, periodicals, wiley, mater, biomed, j, res, inc, part, elsevier, v, environ, toxicol, published
9	chem, environ, toxicol, periodicals, wiley, inc, int, c, j, water, authors, author, assess, neem, agnps
10	nanoparticles, study, silver, antibacterial, activity, oxide, nps, present, antimicrobial, synthesis, based, effects, extract, applications, work
11	authors, c, author, ltd, degrees, inc, results, g, showed, saab, h, society, copyright, study, j
12	activity, antibacterial, results, showed, properties, potential, nps, high, ag, also, compared, applications, study, effect, exhibited
13	chem, nanoparticles, antibacterial, authors, aureus, staphylococcus, activity, escherichia, electron, x, ray, diffraction, scanning, microscopy, infrared
14	nanoparticles, antibacterial, aureus, staphylococcus, authors, activity, environ, escherichia, electron, toxicol, x, ray, diffraction, scanning, microscopy
15	using, study, antibacterial, method, authors, staphylococcus, escherichia, synthesized, present, properties, synthesis, investigated, prepared, based, aureus
16	author, c, using, ltd, antibacterial, inc, staphylococcus, aureus, study, copyright, escherichia, method, degrees, synthesized, society
17	antibacterial, e, study, coli, properties, used, ag, surface, based, potential, nps, applications, present, water, effect
18	toxicol, inc, periodicals, wiley, study, ltd, j, antibacterial, e, elsevier, nps, coli, results, c, properties
19	activity, e, using, coli, nanoparticles, g, dot, center, b, photocatalytic, atcc, ml, showed, method, h
20	study, activity, antimicrobial, nps, e, coli, used, present, x, g, electron, ray, diffraction, center, dot
21	nps, ag, zno, g, using, mu, nm, concentration, ml, respectively, tio2, mg, antibacterial, synthesized, l
22	ltd, elsevier, inc, c, gmbh, j, periodicals, wiley, nps, masson, sas, coli, ireland, techna, e
23	results, study, nps, coli, showed, activity, escherichia, using, zno, ag, author, staphylococcus, aureus, authors, present

Topic number	tokens with most occurrences
24	bacteria, results, nps, negative, antimicrobial, activity, positive, used, coli, surface, gram, based, potential, bacterial, published
25	compos, study, hemocompatibility, compressive, bacteria, cytocompatibility, antibacterial, hydrogels, showed, pul, g, present, negative, tests, positive
26	showed, bacteria, study, negative, positive, using, antibacterial, nps, e, gram, nanoparticles, published, analysis, ltd, author
27	biotechnol, engineers, institute, american, c, chemical, bacteria, j, inc, taiwan, institution, society, showed, periodicals, wiley
28	surface, showed, ag, properties, antimicrobial, silver, published, escherichia, agnps, coli, used, based, bacterial, high, nm
29	published, author, authors, ltd, bacterial, dot, cell, center, cells, biol, elsevier, behalf, escherichia, nps, nanoparticles
30	bioeng, published, al, g, ag, vinasse, cane, silver, mu, bacteria, ml, agnps, author, ltd, authors
31	ag, silver, published, g, agnps, bacteria, mu, ml, e, concentration, results, author, antimicrobial, authors, ltd
32	biol, ag, silver, agnps, g, e, bacteria, mu, ml, published, concentration, results, aureus, respectively, antimicrobial
33	dot, center, escherichia, ag, coli, silver, showed, used, synthesized, biol, method, acid, bacteria, oh, agnps
34	inc, j, periodicals, wiley, published, c, surface, res, mater, behalf, elsevier, escherichia, sas, biomed, masson
35	properties, antimicrobial, e, used, dot, center, showed, aureus, potential, based, published, nps, method, biol, applications
36	bacterial, ag, e, silver, aureus, dot, cell, cells, center, agnps, growth, p, strains, species, also
37	surface, dot, aureus, center, e, study, bacteria, staphylococcus, nm, ag, activity, p, size, area, biol
38	conclusions, outlook, survival, salm, surface, properties, escherichia, g, coli, nm, mu, drawn, size, ml, showed
39	used, method, nm, based, aureus, high, synthesized, showed, size, methods, potential, staphylococcus, agnps, surface, e
40	potential, nm, agnps, size, synthesized, silver, high, average, nps, zeta, particle, applications, spherical, uv, antimicrobial
41	bacterial, surface, used, synthesized, g, agnps, antimicrobial, dot, center, nps, silver, mu, method, ml, showed
42	based, high, bacterial, method, ag, prepared, synthesized, g, showed, methods, water, detection, oxide, novel, low
43	potential, surface, g, ag, antimicrobial, dot, center, mu, using, used, ml, applications, showed, zeta, nanoparticles
44	nm, ag, bacterial, size, used, high, properties, using, study, average, nanoparticles, particle, light, respectively, activity
45	high, silver, agnps, nps, low, bacterial, effect, concentration, efficiency, l, water, due, using, mg, concentrations
46	methods, high, silver, materials, different, concentration, effect, effects, surface, compared, low, nps, concentrations, potential, l

Topic number	tokens with most occurrences
47	high, cell, cells, methods, synthesized, g, nm, properties, method, mu, dot, center, ml, authors, author
48	synthesized, method, high, also, surface, zno, characterized, c, published, ag, negative, different, h, positive, p
49	typhimurium, high, cells, cell, properties, salmonella, synthesized, respectively, methods, j, silver, g, dot, authors, author
50	antimicrobial, effect, antibacterial, nm, method, concentration, cell, activities, high, size, cells, zno, negative, positive, typhimurium
51	j, also, ltd, authors, author, b, v, method, effect, periodicals, wiley, concentration, agnps, different, compared
52	also, g, inc, mu, c, nm, ml, methods, society, antimicrobial, typhimurium, sas, masson, based, method
53	method, silver, prepared, potential, cells, negative, synthesis, positive, well, electron, x, microscopy, different, diffusion, via
54	cells, concentration, effect, respectively, synthesized, bacteria, method, inc, compared, inhibitory, based, mg, methods, low, detection
55	j, cells, c, h, society, g, silver, published, ltd, antimicrobial, degrees, analysis, japan, saab, antibacterial
56	respectively, cell, concentration, also, method, antimicrobial, j, bacteria, compared, h, methods, c, coli, inhibition, p
57	effect, silver, bacteria, synthesized, concentration, j, aureus, cell, light, coli, uv, potential, nm, method, c
58	agnps, effect, c, j, method, society, ag, concentration, published, negative, based, positive, degrees, japan, nanoparticles
59	natl, acad, agnps, different, l, bacteria, antimicrobial, water, prepared, inc, method, mg, aureus, effects, light
60	negative, positive, silver, effect, concentration, gram, based, synthesized, c, society, p, potential, also, aeruginosa, properties
61	concentration, different, also, l, mg, analysis, c, society, high, cell, j, based, inhibitory, concentrations, minimum
62	acad, l, cell, mg, also, proc, concentration, h, water, usa, mechanism, fcrn, show, staphylococcus, escherichia
63	l, mg, h, negative, positive, water, nm, gram, inc, compared, e, effects, exposure, methods, synthesized
64	analysis, revealed, p, compared, confirmed, h, g, method, presence, concentration, significantly, protein, well, structure, expression
65	effect, l, analysis, mg, respectively, h, method, agnps, also, high, potential, properties, positive, bacterial, negative
66	h, uv, light, concentration, zno, prepared, analysis, negative, visible, positive, water, vis, agnps, respectively, time
67	zno, effects, respectively, oxide, species, growth, analysis, toxicity, society, oxygen, l, reactive, zinc, investigated, ros
68	zno, prepared, oxide, water, compared, p, l, analysis, zinc, society, different, mg, cell, cells, agnps
69	h, p, zno, aeruginosa, growth, oxide, based, ramaswamy, high, communicated, pseudomonas, potential, zinc, silver, observed

Topic number	tokens with most occurrences
70	water, h, electron, x, effects, microscopy, scanning, synthesized, ray, treatment, zno, agnps, size, effect, toxicity
71	p, background, aeruginosa, water, pseudomonas, acid, species, prepared, well, society, oxygen, subtilis, inc, concentration, reactive
72	background, compared, h, zno, masson, sas, different, higher, control, staphylococcus, method, positive, e, significantly, x
73	masson, sas, c, j, gmbh, species, degrees, oxygen, l, saab, reactive, p, academic, light, press
74	effects, prepared, uv, growth, characterized, xrd, vis, masson, sas, cell, ftir, tem, toxicity, acid, ir
75	water, method, uv, zno, analysis, p, light, based, effects, visible, masson, sas, different, background, society
76	society, masson, sas, h, japan, elsevier, korean, authors, chemistry, author, industrial, engineering, powder, technology, objective
77	objective, purpose, uv, method, synthesis, water, c, vis, visible, different, compared, degrees, present, treatment, cell
78	prepared, electron, species, microscopy, analysis, oxygen, effects, water, reactive, synthesized, objective, based, scanning, growth, potential
79	purpose, growth, electron, microscopy, light, scanning, inhibition, analysis, time, conclusion, sem, could, energy, treatment, visible
80	purpose, x, species, ray, oxygen, reactive, compared, effects, ros, toxicity, diffraction, prepared, water, two, zno
81	growth, inhibition, species, oxygen, water, x, reactive, purpose, xrd, ray, well, ros, uv, concentrations, increased
82	biochem, et, al, inc, bioinformatics, gmbh, recent, elsevier, ical, sem, size, well, oscillation, h, tem
83	conclusion, prepared, could, different, species, oxygen, coli, reactive, analysis, concentrations, treatment, ml, p, negative, x
84	x, light, growth, ray, nm, analysis, could, effects, visible, min, ml, irradiation, p, photocatalytic, different
85	two, conclusion, growth, acid, compared, e, staphylococcus, escherichia, one, concentration, nm, water, uv, investigated, potential
86	toxicity, size, two, light, prepared, well, x, tio2, ray, observed, found, could, visible, particle, efficiency
87	two, toxicity, could, coli, nm, low, observed, aureus, treatment, ml, concentrations, mu, positive, negative, prepared
88	size, two, coli, aureus, found, time, effects, particle, negative, positive, synthesis, structure, tem, xrd, growth
89	well, could, two, zno, release, effects, treatment, drug, compared, characterized, mg, sem, ml, species, wound
90	well, acid, observed, coli, aureus, presence, protein, increased, zno, mu, positive, x, light, ml, concentrations
91	well, synthesis, oxide, engineers, chemical, coli, compared, aureus, institute, institution, taiwan, applications, extract, growth, green
92	observed, time, significant, prepared, ml, mm, detection, synthesis, concentrations, mg, staphylococcus, e, treatment, extract, first

Topic number	tokens with most occurrences
93	engineers, chemical, institute, institution, taiwan, observed, treatment, american, acid, could, physics, zno, staphylococcus, escherichia, significant
94	treatment, g, concentration, increased, university, well, observed, tio2, saud, king, behalf, oxide, l, applications, significantly
95	university, saud, king, behalf, acid, hosting, ml, could, engineers, masson, sas, size, detection, chemical, mm
96	could, observed, synthesis, acid, effects, applications, oxide, compared, found, structure, extract, green, academic, h, press
97	time, could, g, first, increased, nm, ph, p, synthesis, investigated, observed, exposure, university, protein, escherichia
98	found, increased, well, effective, prepared, protein, significantly, effects, infrared, nm, concentrations, fourier, transform, engineers, tio2
99	release, observed, found, drug, efficiency, ph, g, infrared, fourier, toxicity, encapsulation, transform, exhibited, inhibition, coli
100	mm, oxide, increased, gmbh, efficiency, significantly, p, higher, exhibited, tio2, release, mg, graphene, sem, membrane

Table S 5: Relation between the topics identified via LDA at the abstract level and the 15 most frequent tokens associated to each topic

Topic number	tokens with most occurrences
1	sequence, blot, dot, gene, protein, dna, expressed, cloned, sequences, expression, amino, proteins, escherichia, genes, purified
2	infections, detection, health, resistant, bacterial, ml, antibiotic, antibiotics, pathogens, samples, resistance, study, bacteria, pathogenic, coli
3	surface, escherichia, bacteria, aureus, coli, study, staphylococcus, bacterial, nanoparticles, toxicity, antibacterial, potential, resistant, cells, nm
4	uv, xrd, extract, synthesized, size, spectroscopy, nm, tem, diffraction, fourier, sem, synthesis, ray, spherical, green
5	complexes, ii, ligand, crystal, center, n, complex, ir, dot, compounds, elemental, nmr, coordination, two, ligands
6	dot, dna, gene, center, c, sequence, per, chromosome, show, protein, similar, associated, total, using, elsevier
7	vitro, wound, release, mechanical, adhesion, improved, healing, coating, infection, hydrogel, strength, antibacterial, composite, bone, aureus
8	dot, center, l, study, ml, antioxidant, extract, results, activity, radical, effect, treatment, mg, concentration, respectively
9	new, different, due, developed, one, form, using, significant, specific, high, also, increased, p, reaction, two
10	detection, limit, ml, carbon, time, bacteria, sensitivity, study, showed, cfu, concentration, high, range, dna, cells
11	microscopy, xrd, ray, prepared, sem, analysis, antibacterial, spectroscopy, synthesized, uv, efficiency, also, x, tem, ftir
12	exposure, toxicity, effects, observed, concentrations, exposed, l, escherichia, mg, induced, results, zebrafish, nanoparticles, study, stress
13	photocatalytic, light, degradation, environmental, visible, mechanism, irradiation, fluorescence, dot, species, applications, carbon, also, one, samples
14	toxicity, exposure, aquatic, toxic, effects, exposed, concentrations, organisms, study, nanoparticles, results, mg, l, used, concentration
15	detection, fluorescence, fluorescent, limit, cells, signal, cfu, method, vivo, assay, ml, dna, specific, quantum, high
16	fish, kg, fed, immune, diet, levels, groups, dietary, diets, serum, control, significantly, liver, group, feed
17	dot, acid, fabrics, cotton, effects, center, used, microbial, found, higher, different, samples, ph, role, analysis
18	properties, excellent, aureus, antibacterial, exhibited, furthermore, applications, e, activity, materials, good, resistant, bacterial, surface, composite
19	exposure, exposed, toxicity, aquatic, fish, effects, zebrafish, organisms, mg, concentrations, l, observed, toxic, study, stress

Topic number	tokens with most occurrences
20	light, irradiation, visible, xrd, uv, photocatalytic, blue, vis, degradation, diffraction, nm, sem, efficient, bacteria, spectroscopy
21	ray, electron, diffraction, x, antibacterial, spectroscopy, scanning, microscopy, uv, nanoparticles, synthesized, prepared, activity, xrd, surface
22	extract, ftir, electron, xrd, fourier, nanoparticles, antimicrobial, size, spectroscopy, uv, green, transmission, synthesized, characterized, transform
23	infections, biofilm, wound, staphylococcus, aureus, resistant, release, antibacterial, antimicrobial, antibiotic, activity, drug, bacterial, treatment, results
24	photocatalytic, light, degradation, water, efficiency, antibacterial, performance, due, irradiation, based, surface, materials, synthesized, composite, oxygen
25	infection, infections, vivo, strategy, therapy, infected, resistance, bacterial, vitro, antibacterial, release, healing, wound, resistant, antibiotic
26	increased, significantly, decreased, kg, p, treatment, levels, treated, weight, control, stress, days, higher, increase, significant
27	ray, fabric, surface, properties, coli, fabrics, aureus, spectroscopy, electron, antibacterial, activity, cotton, x, prepared, nanoparticles
28	growth, center, production, l, g, dot, weight, biomass, content, ratio, levels, fed, kg, control, total
29	photocatalytic, degradation, visible, light, xrd, min, dye, wastewater, efficiency, photocatalyst, doped, uv, removal, elsevier, blue
30	detection, limit, sensitivity, sensitive, gold, cfu, ml, based, signal, samples, sensor, range, linear, sensing, biosensor
31	toxicity, exposure, aquatic, exposed, effects, organisms, environmental, l, concentrations, nps, nanoparticles, soil, different, reserved, daphnia
32	films, coli, properties, film, mechanical, packaging, prepared, antibacterial, food, activity, based, showed, aureus, nanoparticles, water
33	performance, water, properties, excellent, work, contact, coli, process, antibacterial, applications, high, composite, surface, efficiency, h
34	wound, healing, tissue, wounds, loaded, infection, skin, release, vivo, hydrogel, dressing, vitro, proliferation, inflammatory, treatment
35	model, detection, nanoparticles, fluorescence, strategy, cell, effective, clinical, species, bacterial, treatment, anti, novel, zebrafish, fluorescent
36	photocatalytic, light, oxygen, reactive, species, min, efficiency, visible, irradiation, performance, coli, degradation, disinfection, water
37	including, health, resistant, bacterial, infections, antibiotic, reactive, antibiotics, thus, staphylococcus, cell, cells, resistance, aureus, antimicrobial
38	cells, molecular, protein, nanoparticles, interactions, however, mechanism, development, cell, formation, complex, cellular, effects, membrane, functional

Topic number	tokens with most occurrences
39	resistant, antibiotic, staphylococcus, resistance, antimicrobial, positive, infections, activity, inhibitory, antibiotics, antibacterial, strains, aureus, center, aeruginosa
40	genes, expression, stress, dot, gene, proteins, protein, induced, regulation, mechanisms, however, molecular, involved, related, intracellular
41	electron, synthesized, spectroscopy, ray, microscopy, size, activity, coli, antibacterial, x, nanoparticles, synthesis, escherichia, diffraction, used
42	ray, scanning, x, electron, spectroscopy, microscopy, antibacterial, diffraction, aureus, properties, sem, escherichia, coli, staphylococcus, prepared
43	adhesion, coating, implants, bone, layer, coatings, implant, surfaces, infection, surface, aureus, titanium, properties, biocompatibility, antibacterial
44	bone, implants, proliferation, implant, cells, aureus, vitro, infection, titanium, adhesion, coating, coatings, clinical, cell, biocompatibility
45	reserved, e, rights, results, mg, used, species, elegans, stress, showed, exposure, oxidative, elsevier, caenorhabditis, properties
46	nanoparticles, escherichia, e, coli, aureus, properties, method, antibacterial, reserved, prepared, sem, showed, composite, rights, cells
47	exposure, toxicity, organisms, aquatic, effects, exposed, zebrafish, toxic, observed, concentrations, environment, results, nanomaterials, concentration, staphylococcus
48	dot, binding, protein, dna, blot, center, enzyme, sequence, proteins, specific, site, recombinant, expressed, two, expression
49	exposure, exposed, toxicity, organisms, effects, aquatic, oxidative, increased, stress, mg, significantly, study, toxic, however, escherichia
50	films, mechanical, properties, coli, aureus, film, polymer, packaging, prepared, antibacterial, antimicrobial, strength, staphylococcus, surface, thermal
51	cell, however, membrane, coli, using, induced, structure, cellular, results, also, study, species, cells, bacterial, mechanisms
52	published, open, article, cc, access, license, http, org, creativecommons, licenses, nd, behalf, university, stress, saud
53	gene, genes, expression, dot, protein, center, involved, plant, arabidopsis, plants, proteins, expressed, species, function, mechanisms
54	xrd, uv, ray, spectroscopy, diffraction, synthesis, electron, nm, extract, x, antibacterial, fourier, synthesized, nanoparticles, aureus
55	infections, infection, drug, therapy, vivo, wound, antibiotic, bacterial, healing, aureus, clinical, resistant, delivery, resistance, antibiotics
56	extract, synthesis, uv, green, spherical, xrd, synthesized, antioxidant, nm, nanoparticles, antibacterial, tem, agnps, size, ug
57	protein, enzyme, c, recombinant, nanoparticles, reserved, microscopy, elsevier, rights, purified, using, particles, binding, acid, l

Topic number	tokens with most occurrences
58	ray, diffraction, x, electron, spectroscopy, xrd, microscopy, uv, synthesized, method, aureus, sem, scanning, coli, activity
59	cfu, ml, antimicrobial, used, escherichia, l, detection, food, methods, showed, coli, pseudomonas, concentration, aureus, nm
60	center, energy, protein, transfer, dot, reaction, well, enzyme, mechanism, molecular, based, free, theory, structural, binding
61	extract, nm, uv, size, synthesis, spherical, synthesized, green, silver, xrd, electron, ray, using, spectroscopy, vis
62	zebrafish, toxicity, exposure, embryos, exposed, effects, toxic, aquatic, concentrations, organisms, larvae, study, nm, using, nps
63	radical, center, beta, dot, class, complex, site, alpha, protein, subunit, reduction, pathway, two, ribonucleotide, important
64	xrd, ray, diffraction, uv, spectroscopy, x, synthesized, sem, scanning, electron, visible, photocatalytic, nm, antibacterial, fourier
65	complexes, center, dot, ii, elemental, ir, h2o, ligand, complex, crystal, nmr, h, ligands, n, cu
66	dot, use, however, methods, center, infections, compared, used, delivery, formation, results, study, antimicrobial, cell, thus
67	detection, limit, cfu, magnetic, ml, min, protein, high, method, based, time, escherichia, using, separation, also
68	detection, limit, cfu, ml, escherichia, sensitivity, based, coli, using, sensitive, samples, e, carbon, method, used
69	bacterial, antibacterial, staphylococcus, bacteria, aureus, promising, coli, surface, due, potential, health, infections, release, negative, properties
70	wound, mechanical, antioxidant, fourier, transform, properties, antimicrobial, activity, aureus, inhibition, scanning, silver, antibacterial, extract, nm
71	therapy, drug, infections, vivo, resistant, release, infection, delivery, cells, loaded, efficacy, vitro, therapeutic, treatment, bacterial
72	packaging, mechanical, properties, food, antibacterial, films, coli, aureus, prepared, loaded, release, escherichia, scanning, activity, poly
73	elsevier, c, silver, nanoparticles, study, toxicity, coli, potential, effects, electron, applications, using, used, observed, escherichia
74	used, air, c, conditions, particles, compounds, performance, environmental, mg, rights, efficiency, structure, h, l, also
75	protein, binding, membrane, proteins, cell, expression, dot, cells, cellular, molecules, specific, model, high, analysis, dependent
76	cells, delivery, cell, nanoparticles, results, vivo, toxicity, using, drug, used, efficacy, zebrafish, thus, bacteria, silver

Topic number	tokens with most occurrences
77	surface, antibacterial, properties, contact, water, materials, e, performance, scanning, composite, rights, study, spectroscopy, ray, microscopy
78	aureus, staphylococcus, antimicrobial, nm, size, microscopy, characterized, bacteria, sem, antibacterial, using, activity, silver, scanning, spectroscopy
79	characterized, synthesized, spectroscopy, ray, center, analysis, c, rights, uv, ir, x, dot, coli, metal, infrared
80	ray, xrd, antibacterial, diffraction, bacteria, x, staphylococcus, electron, spectroscopy, toxicity, nanoparticles, synthesis, coli, escherichia, microscopy
81	films, rights, c, water, escherichia, reserved, properties, different, scanning, solution, surface, investigated, electron, prepared, elsevier
82	genes, dot, gene, center, higher, however, increased, expression, study, chromosome, system, species, levels, found, analysis
83	aureus, graphene, coli, go, membrane, oxide, surface, antibacterial, staphylococcus, study, prepared, escherichia, water, activity, showed
84	stress, plants, superoxide, oxidative, dismutase, plant, genes, antioxidant, center, expression, catalase, sod, peroxidase, levels, reactive
85	protein, recombinant, proteins, expressed, gene, expression, purified, major, immune, virus, binding, type, high, two, vaccine
86	center, dot, reactive, water, l, toxicity, oh, treatment, removal, species, environmental, results, mg, ph, radicals
87	detection, limit, sensitive, sensitivity, cfu, gold, samples, electrochemical, rapid, electrode, linear, detect, probe, ml, specific
88	stress, oxidative, superoxide, induced, dismutase, expression, genes, catalase, gene, exposure, l, significantly, levels, exposed, toxicity
89	extract, uv, synthesis, synthesized, spectroscopy, nanoparticles, antimicrobial, silver, nm, ray, fourier, size, antioxidant, activity, spherical
90	resistant, therapy, infections, vivo, infection, drug, photothermal, bacteria, treatment, bacterial, reactive, staphylococcus, provides, based, development
91	ray, x, electron, nanoparticles, antibacterial, spectroscopy, diffraction, microscopy, scanning, ag, synthesized, fourier, silver, properties, coli
92	detection, rapid, limit, cfu, method, detect, methods, samples, ml, sensitivity, food, based, gold, pathogens, developed
93	proteins, assay, protein, human, cells, cancer, cell, combination, anti, study, methods, infrared, resistant, using, nanoparticles
94	staphylococcus, wound, aureus, antibacterial, release, activity, coli, prepared, properties, showed, loaded, scanning, poly, results, microscopy
95	center, dot, values, total, complexes, c, respectively, yield, value, well, determined, l, three, h, production
96	antimicrobial, inhibitory, aureus, drug, staphylococcus, antibacterial, mic, results, nanoparticles, study, antibiotic, bacterial, resistant, vitro, aeruginosa

Topic number	tokens with most occurrences
97	dna, single, protein, molecular, biological, cells, detection, complex, high, used, based, presence, specific, well, probe
98	diffraction, x, ray, xrd, method, scanning, uv, synthesized, sem, photocatalytic, spectroscopy, properties, antibacterial, structure, electron
99	protein, expressed, proteins, gene, recombinant, binding, expression, blot, dot, sequence, specific, virus, purified, fusion, identified
100	ray, x, spectroscopy, inhibition, results, synthesized, showed, uv, method, scanning, photocatalytic, zno, electron, materials, fourier

Table S 6: Relation between the topics identified via LDA at the sentence level and the 15 most frequent tokens associated to each topic

Topic number	tokens with most occurrences
1	c, reserved, rights, elsevier, b, v, ltd, published, inc, acta, materialia, authors, copyright, sas, science
2	carbon, nanotubes, sio2, nanoparticles, surface, mesoporous, walled, functionalized, mwcnts, multi, single, cnts, nanotube, loaded, modified
3	c, degrees, temperature, high, h, ph, weight, low, molecular, conditions, water, temperatures, solution, stable, room
4	fluorescence, emission, quantum, two, ii, center, dot, nm, electron, n, dots, state, excitation, oxygen, transfer
5	surface, concentration, charged, size, increased, increase, due, positively, charge, increasing, negatively, ph, higher, observed, adsorption
6	nanoparticles, extract, using, silver, study, synthesis, green, agnps, synthesized, nps, present, leaf, aqueous, method, reducing
7	protein, expression, immune, alpha, il, dot, mice, cells, specific, beta, blot, response, antibodies, antibody, anti
8	study, toxicity, effects, nps, different, nanoparticles, magna, investigated, daphnia, using, present, two, exposure, tio2, model
9	degradation, photocatalytic, dye, blue, methylene, activity, light, efficiency, min, mb, irradiation, removal, catalytic, visible, dyes
10	properties, investigated, chemical, thermal, antibacterial, structural, characterized, studied, different, mechanical, analysis, optical, using, mass, morphological
11	l, mg, concentrations, exposure, toxicity, nps, concentration, h, exposed, observed, effects, ag, g, toxic, showed
12	delivery, drug, therapy, potential, treatment, promising, system, therapeutic, bacterial, infections, based, cancer, strategy, effective, study
13	high, water, new, method, based, cost, low, provides, work, efficient, applications, application, strategy, study, potential
14	nm, electron, sem, microscopy, size, spherical, tem, showed, nanoparticles, images, scanning, revealed, transmission, morphology, surface
15	nanoparticles, chitosan, films, prepared, silver, study, pva, cellulose, based, using, method, zno, nanocomposite, composite, ag
16	disease, diseases, human, water, pathogens, food, important, virus, one, health, bacteria, used, control, cause, infectious
17	c, b, v, elsevier, published, society, behalf, university, graphics, king, subtilis, j, saud, author, chemical
18	antibacterial, properties, nanoparticles, silver, ag, surface, activity, graphene, oxide, antimicrobial, go, coating, effect, coatings, composite
19	detection, ml, limit, cfu, e, coli, range, samples, sensitivity, concentration, linear, x, low, method, high

Topic number	tokens with most occurrences
20	graphene, oxide, method, synthesized, go, doped, prepared, using, nanoparticles, carbon, tio2, rgo, study, via, ag
21	molecular, interactions, binding, interaction, analysis, model, mechanism, structure, using, docking, experimental, studies, results, hydrogen, theory
22	l, g, ph, concentration, time, mg, adsorption, conditions, order, model, parameters, rate, using, maximum, respectively
23	fish, control, groups, group, mg, liver, p, trout, kg, significantly, rainbow, compared, levels, study, days
24	nm, size, average, spherical, particle, nanoparticles, nps, agnps, shape, potential, diameter, zeta, synthesized, respectively, results
25	coli, gene, protein, escherichia, e, expression, genes, type, strain, expressed, plasmid, sequence, recombinant, proteins, amino
26	metal, ions, organic, groups, nanoparticles, surface, magnetic, adsorption, fe3o4, iron, framework, ii, reaction, immobilized, acid
27	however, toxicity, effects, nanoparticles, still, nps, studies, mechanisms, organisms, mechanism, known, remains, although, aquatic, toxic
28	genes, expression, gene, analysis, pcr, related, identified, revealed, expressed, time, dot, using, involved, response, sequencing
29	membrane, membranes, surface, compared, cell, flux, water, adhesion, ag, proliferation, cells, showed, coating, serum, albumin
30	cell, membrane, bacterial, damage, cells, bacteria, wall, mechanism, species, ros, oxygen, reactive, death, intracellular, leakage
31	dna, dot, protein, binding, center, complex, two, structure, c, site, sequence, proteins, base, beta, residues
32	analysis, xrd, confirmed, ftir, x, ray, diffraction, structure, groups, presence, functional, spectroscopy, infrared, crystalline, revealed
33	uv, nm, vis, absorption, visible, peak, surface, band, plasmon, resonance, nanoparticles, agnps, spectroscopy, spectra, showed
34	aureus, biofilm, antibacterial, staphylococcus, activity, coli, bacterial, using, method, growth, diffusion, aeruginosa, bacteria, formation, e
35	ml, g, concentration, mu, mic, mg, inhibitory, minimum, respectively, l, e, concentrations, coli, values, aureus
36	plant, growth, plants, root, l, study, arabidopsis, stress, roots, leaves, qds, seed, effects, germination, nps
37	l, solution, ph, water, concentration, concentrations, silver, different, c, ag, mg, mmol, medium, degrees, agno3
38	dna, using, dot, pcr, hybridization, blot, specific, assay, gene, used, reaction, rna, probes, probe, single
39	surface, structure, area, results, size, nanoparticles, zno, g, high, ag, specific, showed, morphology, doping, magnetic
40	light, irradiation, coli, uv, visible, e, inactivation, tio2, min, photocatalytic, within, disinfection, bacteria, dark, escherichia

Topic number	tokens with most occurrences
41	gold, nanoparticles, surface, fluorescence, bacteria, target, magnetic, dna, probe, signal, binding, modified, specific, detection, e
42	resistance, bacteria, resistant, antimicrobial, antibiotic, antibiotics, bacterial, new, antibacterial, development, agents, drug, infections, novel, alternative
43	spectroscopy, uv, x, ray, electron, xrd, characterized, microscopy, vis, diffraction, sem, tem, ftir, infrared, fourier
44	results, potential, study, could, treatment, water, used, promising, antibacterial, findings, suggest, effective, wastewater, applications, application
45	antibacterial, wound, properties, applications, potential, bone, tissue, materials, healing, dressing, promising, material, biocompatibility, used, implants
46	applications, potential, biomedical, properties, antibacterial, materials, nanoparticles, promising, fields, used, great, due, various, biological, antimicrobial
47	vivo, model, zebrafish, vitro, toxicity, using, elegans, study, used, studies, cells, imaging, caenorhabditis, c, models
48	antibacterial, activity, nps, antimicrobial, ag, zno, activities, nanoparticles, results, properties, showed, synthesized, antioxidant, compared, exhibited
49	spectroscopy, electron, x, ray, microscopy, characterized, scanning, infrared, diffraction, fourier, transform, xrd, sem, ftir, analysis
50	method, using, nanoparticles, synthesized, dots, carbon, prepared, silver, ag, synthesis, quantum, one, via, hydrothermal, cds
51	zebrafish, exposure, embryos, effects, toxicity, nps, larvae, development, results, observed, exposed, developmental, fish, induced, changes
52	coli, escherichia, e, bacillus, bacteria, subtilis, strains, salmonella, aureus, isolated, study, staphylococcus, bacterial, strain, b
53	food, packaging, potential, films, used, antimicrobial, active, results, film, life, could, properties, applications, shelf, antibacterial
54	coli, aureus, inhibition, activity, antibacterial, e, mm, zone, staphylococcus, escherichia, concentration, respectively, aeruginosa, showed, inhibitory
55	coli, gram, aureus, negative, positive, antibacterial, bacteria, escherichia, staphylococcus, activity, e, antimicrobial, showed, exhibited, aeruginosa
56	role, important, stress, signaling, response, expression, play, genes, pathway, involved, results, protein, cell, plays, induced
57	acid, acids, increased, fatty, l, production, metabolism, stress, significantly, type, amino, expression, growth, wild, levels
58	wound, healing, vivo, wounds, infected, skin, model, infection, dressing, treatment, hydrogel, dressings, antibacterial, bacterial, tissue
59	dot, time, h, days, c, center, degrees, rate, p, period, compared, storage, increased, higher, significantly

Topic number	tokens with most occurrences
60	water, work, new, materials, efficient, study, friendly, effective, approach, based, antibacterial, provides, cost, method, applications
61	health, resistance, bacteria, bacterial, human, resistant, antibiotic, public, infections, threat, antibiotics, major, serious, global, one
62	acid, chitosan, poly, loaded, nanofibers, prepared, pva, using, nanoparticles, cs, study, electrospinning, electrospun, alcohol, cross
63	release, drug, ph, sustained, loaded, controlled, showed, vitro, h, loading, released, efficiency, encapsulation, profile, rate
64	detection, based, rapid, sensitive, bacteria, method, developed, sers, coli, using, e, biosensor, simple, platform, sensitivity
65	oxygen, species, reactive, ros, generation, production, h2o2, activity, dot, center, hydrogen, peroxide, electron, oxide, oxidation
66	resistant, aureus, activity, mrsa, staphylococcus, antibacterial, methicillin, antibiotic, antibiotics, strains, drug, effect, compared, showed, coli
67	silver, nanoparticles, reducing, agnps, agent, extract, reduction, ag, capping, agents, ions, synthesis, using, used, presence
68	antioxidant, activity, dpph, scavenging, radical, free, acid, g, showed, dot, center, extract, ml, abts, total
69	stability, cycles, excellent, high, water, properties, antibacterial, activity, fabrics, good, exhibited, showed, fabric, performance, cotton
70	cell, cells, cancer, cytotoxicity, ml, human, activity, mu, g, lines, line, assay, cytotoxic, showed, viability
71	light, photothermal, nir, photocatalytic, charge, enhanced, efficiency, effect, near, irradiation, activity, visible, separation, performance, infrared
72	poly, acid, n, surface, polymerization, glycol, synthesized, polymer, groups, via, nanoparticles, prepared, using, co, ethylene
73	surface, using, plasma, surfaces, deposition, layer, air, coating, high, used, filter, water, deposited, film, current
74	nanoparticles, ag, doped, method, zno, synthesized, using, tio2, gel, sio2, nps, prepared, sol, silver, study
75	coli, aureus, e, escherichia, antibacterial, staphylococcus, bacteria, activity, gram, negative, positive, showed, growth, respectively, antimicrobial
76	nanoparticles, study, first, report, gold, antimicrobial, based, antibacterial, work, synthesis, herein, novel, silver, time, present
77	protein, coli, binding, e, proteins, escherichia, recombinant, surface, using, peptide, specific, used, affinity, antibody, self
78	size, nm, particle, nanoparticles, potential, diameter, particles, zeta, average, efficiency, sizes, surface, small, distribution, stability
79	nanoparticles, silver, cotton, antibacterial, ag, method, fabric, fabrics, surface, using, study, coating, fibers, situ, prepared

Topic number	tokens with most occurrences
80	cells, cell, cytotoxicity, human, vitro, biocompatibility, viability, showed, assay, fibroblast, toxicity, fibroblasts, proliferation, good, non
81	analysis, ms, using, mass, different, chromatography, spectrometry, compounds, two, identified, gc, liquid, results, cerevisiae, data
82	bacteria, growth, microbial, cells, bacterial, conditions, may, metal, extracellular, role, also, environment, soil, metals, substances
83	infections, bacterial, infection, aureus, biofilm, staphylococcus, associated, resistant, formation, bacteria, patients, biofilms, cause, treatment, mrsa
84	mg, dot, center, l, kg, p, g, control, significantly, increased, weight, compared, growth, respectively, higher
85	bacterial, infections, treatment, infection, potential, effective, may, antimicrobial, promising, clinical, biofilm, however, associated, therapeutic, use
86	dot, center, oh, species, radicals, degradation, radical, h2o2, hydroxyl, oxygen, reactive, mechanism, process, h, role
87	properties, mechanical, strength, films, water, increased, tensile, film, addition, thermal, improved, showed, stability, composite, results
88	toxicity, study, environmental, potential, effects, aquatic, understanding, nanomaterials, environment, organisms, results, nanoparticles, nps, risk, provide
89	aureus, coli, staphylococcus, escherichia, activity, antibacterial, aeruginosa, pseudomonas, antimicrobial, bacteria, gram, bacillus, albicans, negative, positive
90	cells, bacteria, bacterial, could, biofilm, effectively, cell, biofilms, nps, tumor, irradiation, macrophages, nanoparticles, effect, membrane
91	cell, cells, damage, induced, oxidative, stress, dna, ros, nps, apoptosis, membrane, exposure, results, death, caused
92	cells, cell, fluorescence, microscopy, imaging, fluorescent, membrane, using, protein, confocal, single, dots, time, localization, yeast
93	complexes, ii, center, dot, n, synthesized, complex, ligand, cu, metal, l, co, iii, h2o, h
94	nanoparticles, synthesis, green, silver, friendly, applications, due, properties, metal, nps, used, chemical, agnps, eco, cost
95	however, water, use, environmental, due, environment, used, products, application, nanoparticles, production, applications, high, low, materials
96	antibacterial, coli, e, activity, aureus, ag, showed, escherichia, effect, staphylococcus, silver, antimicrobial, release, bacteria, results
97	increased, activity, significantly, levels, sod, stress, activities, antioxidant, exposure, decreased, superoxide, expression, oxidative, glutathione, cat
98	compared, showed, activity, growth, results, significant, bacterial, higher, significantly, effect, p, inhibition, observed, reduction, concentration
99	study, oil, essential, nanoparticles, antimicrobial, aim, activity, antibacterial, evaluate, present, aimed, investigate, properties, loaded, chitosan

Topic number	tokens with most occurrences
100	microscopy, electron, scanning, sem, using, spectroscopy, characterized, morphology, surface, transmission, analysis, tem, atomic, light, scattering

2.2 Nanomaterials properties

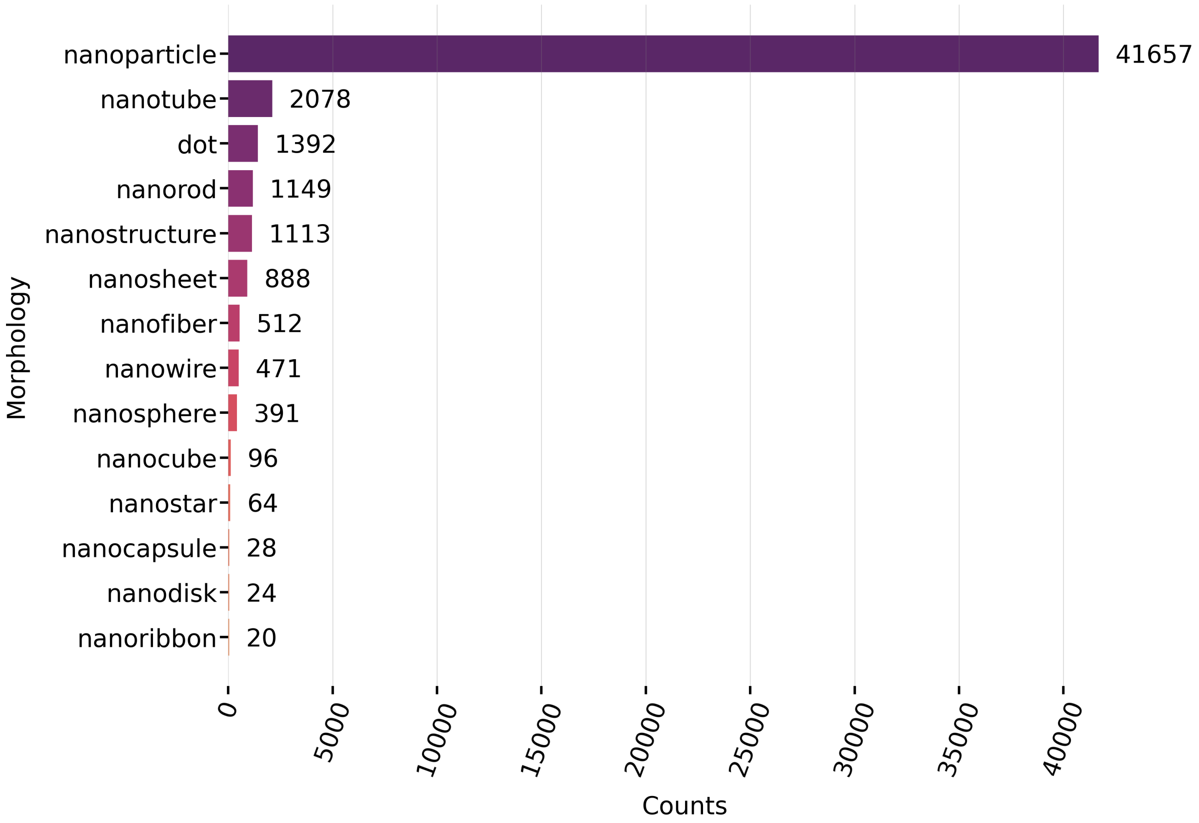

Figure S 3: Frequencies (counts) of terms occurring in the nanotoxicology corpus analyzed. Horizontal bar-plots describe occurrences of terms related to the nanomaterials morphologies used in the studies.

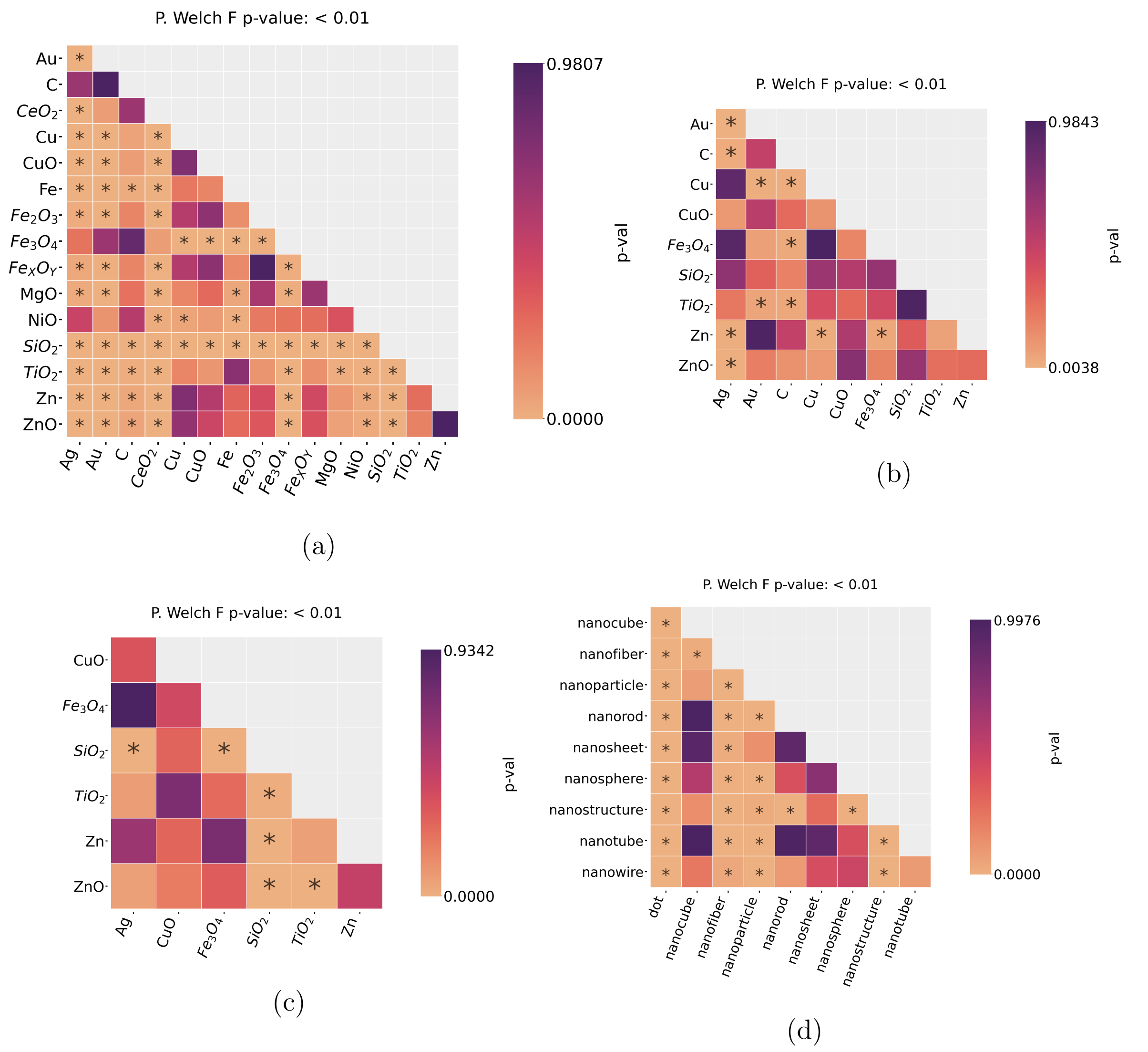

Figure S 4: Pairwise comparison using Welch-studentised mean difference (with 20,000 permutations) for the nanomaterial (S4a) size, (S4b) zeta potential and (S4c) surface area distributions as a function of the nanomaterial composition, and for (S4d) nanomaterials size as a function of the nanomaterial morphology. Pairs with p-values smaller than 0.05 are indicated with asterisks. The result of the global Welch-F test p-value (with 20,000 permutations) is at the top of the graphics.

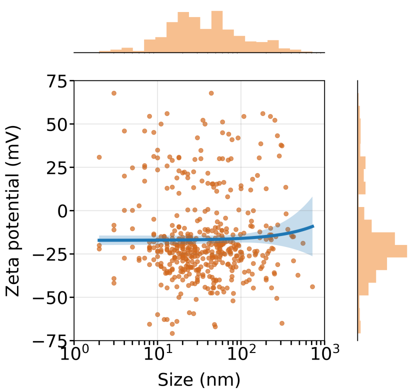

(a)

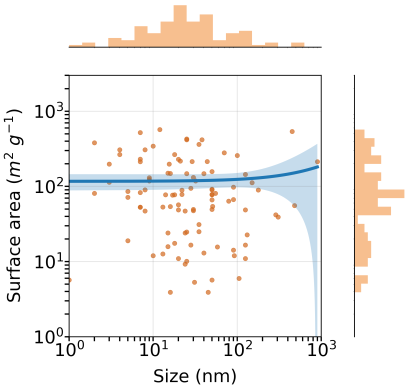

(b)

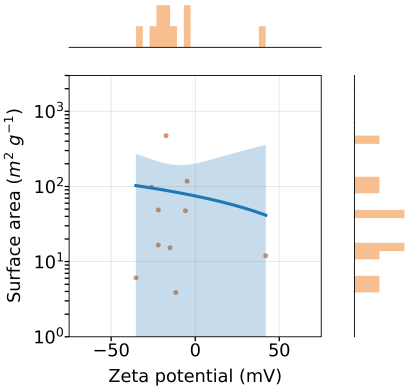

(c)

Figure S 5: Comparison of nanomaterial properties through three scatter plots. Scatter plot of (S5a) nanomaterial size versus zeta potential, (S5b) nanomaterial size versus surface area, and (S5c) zeta potential as a function of surface area. The curve in each panel shows the linear regression fit, and the shaded band indicates the 95% confidence interval (CI).

2.3 Interactions with microorganisms

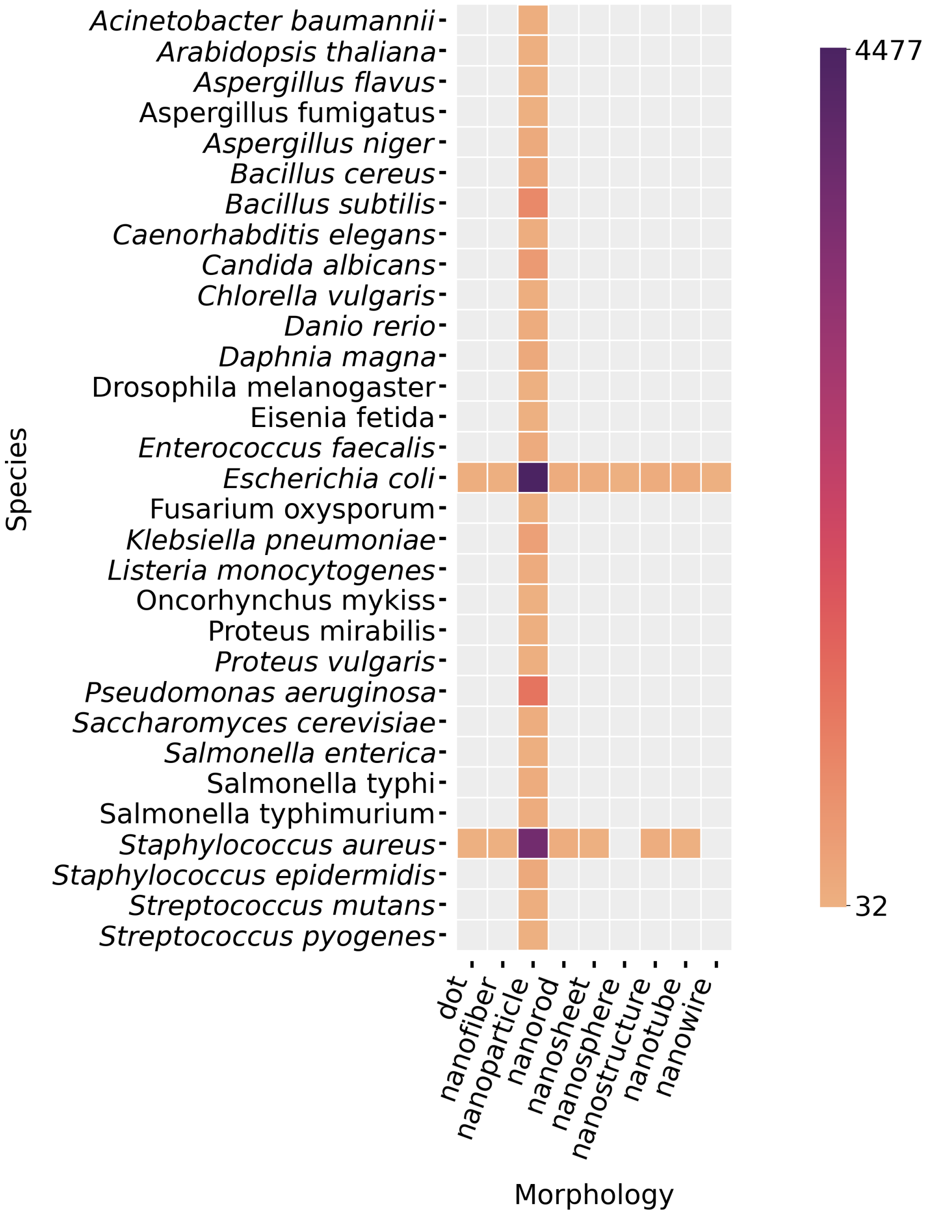

Figure S 6: Frequency distribution (counts) of combined terms occurring in the nanotoxicology corpus analyzed. The correlations between the nanomaterial morphologies with biological organisms was assessed. The color scale represents the occurrence counting, with darker shades indicating higher frequencies.

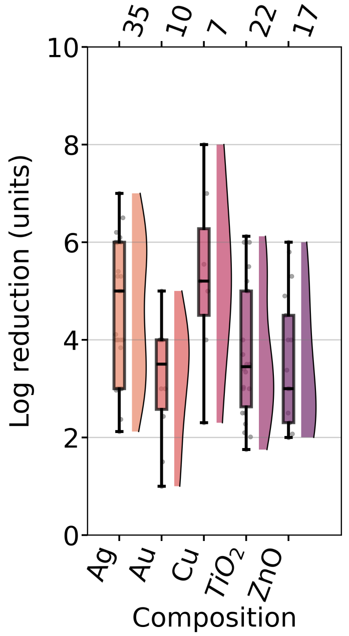

(a)

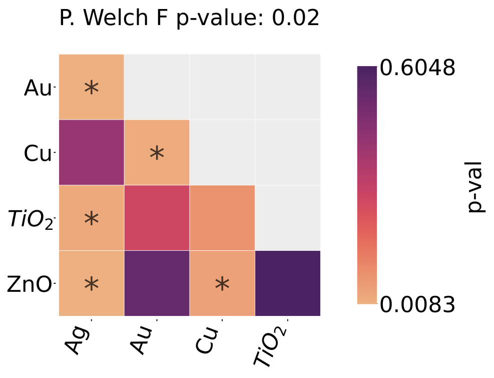

(b)

Figure S 7: (S7a) Box-plot graph representing the relation between the composition and the overall logarithmic reduction (log reduction) of microorganisms considering all species. Numbers at the top of the box-plots indicate how many articles were used in each values distribution. Box-plots were plotted for categories for which at least 7 numerical values were extracted. The distribution function found via kernel density estimator (KDE) for each box-plot is represented at the right side. (S7b) Pairwise comparison using Welch-studentised mean difference (with 20,000 permutations) for the logarithmic reduction as a function of the nanomaterial composition considering all species. Pairs with p-values smaller than 0.05 are indicated with asterisks. The result of the global Welch-F test p-value (with 20,000 permutations) is at the top of the graphics.

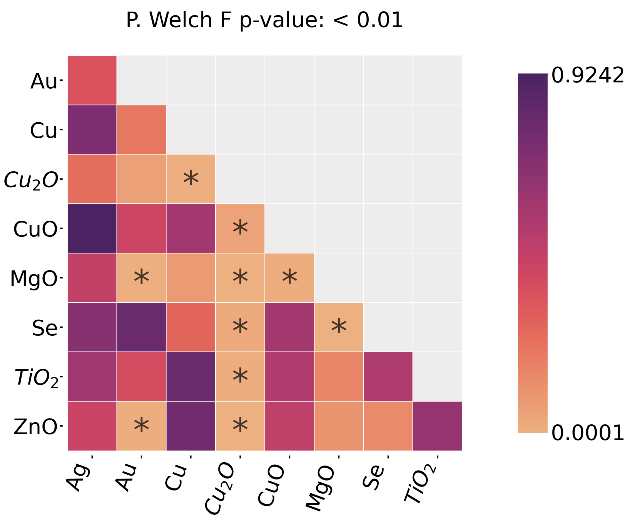

(a)

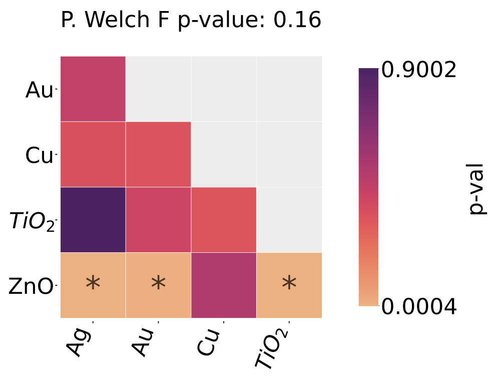

(b)

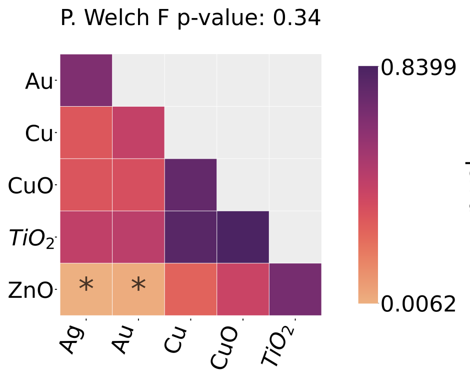

(c)

Figure S 8: Pairwise comparison using Welch-studentised mean difference (with 20,000 permutations) for the minimum inhibitory concentration (MIC) as a function of the nanomaterial composition considering (S8a) all species, and just for (S8b) *Escherichia coli* and (S8c) *Staphylococcus aureus*. Pairs with p-values smaller than 0.05 are indicated with asterisks. The result of the global Welch-F test p-value (with 20,000 permutations) is at the top of the graphics.

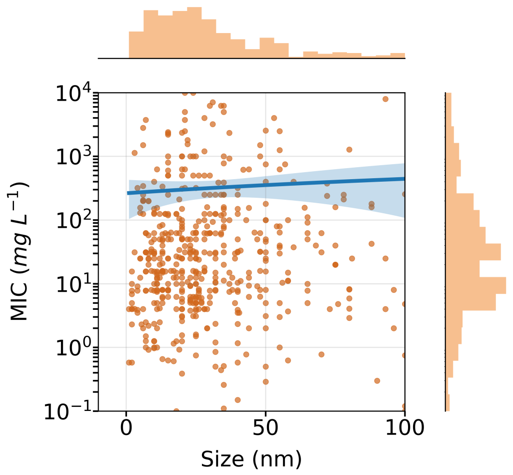

(a)

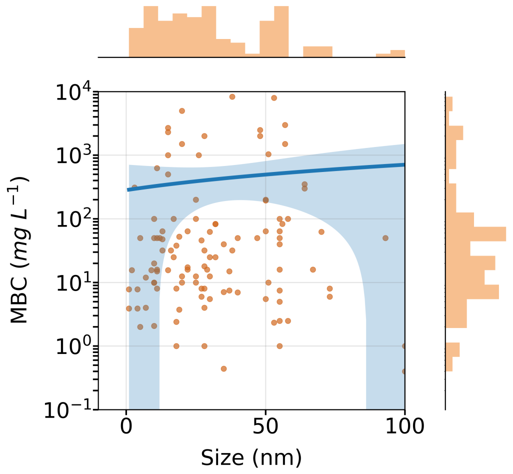

(b)

Figure S 9: Scatter plots showing the nanomaterials size distributions versus (S9a) the minimum inhibitory concentration (MIC) and (S9b) the minimum bactericidal concentration for all species and for all compositions. Histograms representing the values distributions are displayed at the top and right in the graphs. The curve in each panel shows the linear regression fit, and the shaded band indicates the 95% confidence interval (CI).

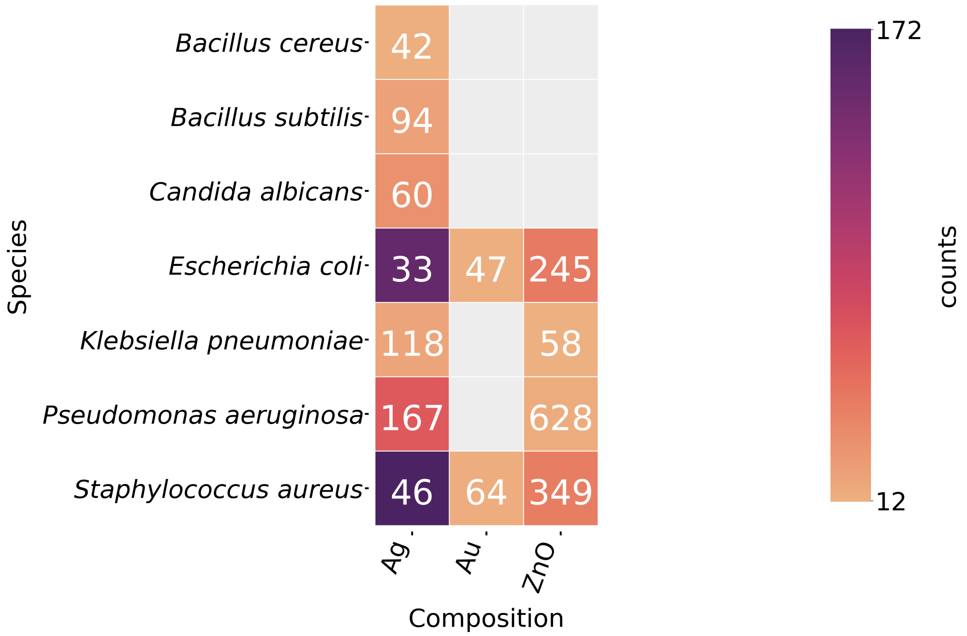

Figure S 10: Heatmap showing mean MIC values as a function of the nanomaterials composition and the biological species tested. The colors in the heatmap indicate the number (counts) of extracted values while the number inside the boxes indicate the mean MIC value.

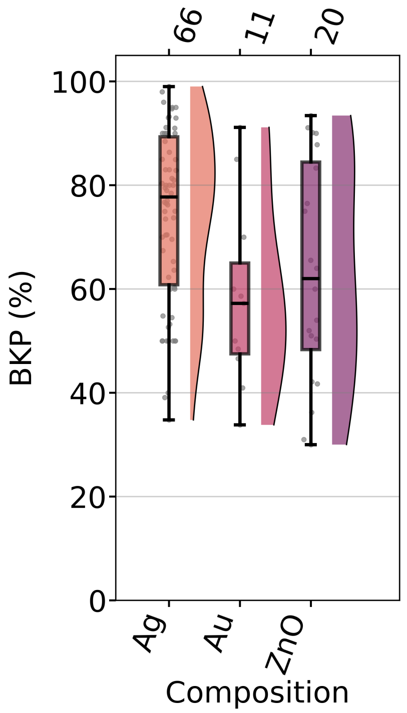

(a)

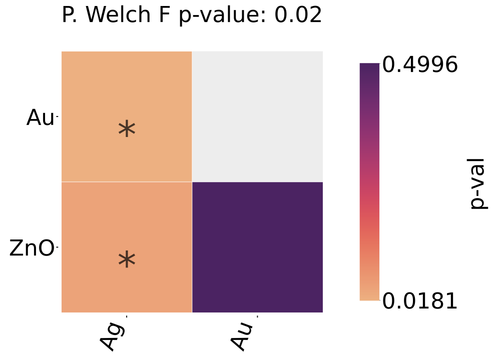

(b)

Figure S 11: (S11a) Box-plot graph representing the relation between the composition and the overall biofilm killing percentage (BKP) of microorganisms considering all species. Numbers at the top of the box-plots indicate how many articles were used in each values distribution. Box-plots were plotted for categories for which at least 7 numerical values were extracted. The distribution function found via kernel density estimator (KDE) for each box-plot is represented at the right side. (S11b) Pairwise comparison using Welch-studentised mean difference (with 20,000 permutations) for the biofilm killing percentage as a function of the nanomaterial composition considering all species. Pairs with p-values smaller than 0.05 are indicated with asterisks. The result of the global Welch-F test p-value (with 20,000 permutations) is at the top of the graphics.

2.4 Interactions with superior organisms

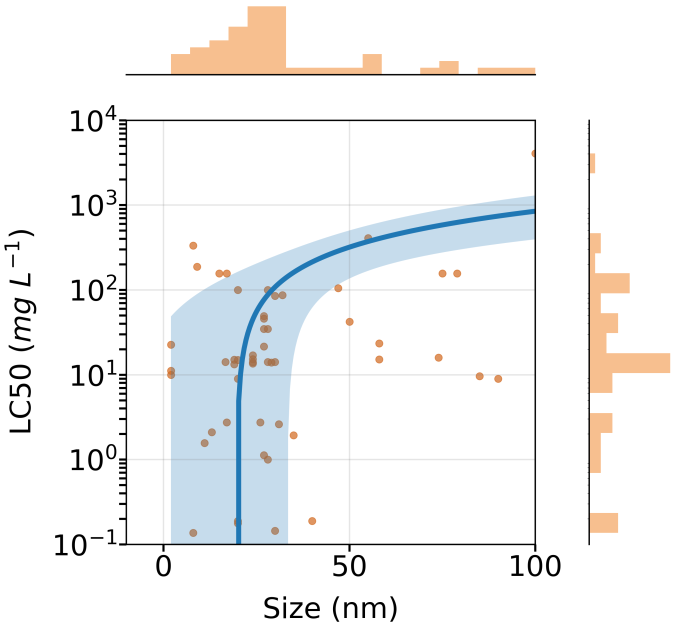

Figure S 12: Scatter plots showing the nanomaterials size distributions versus the all lethal concentration 50% (LC_{50}) extracted for biological species present in the corpus. Histograms representing the values distributions are displayed at the top and right in the graphs. The curve in each panel shows the linear regression fit, and the shaded band indicates the 95% confidence interval (CI).

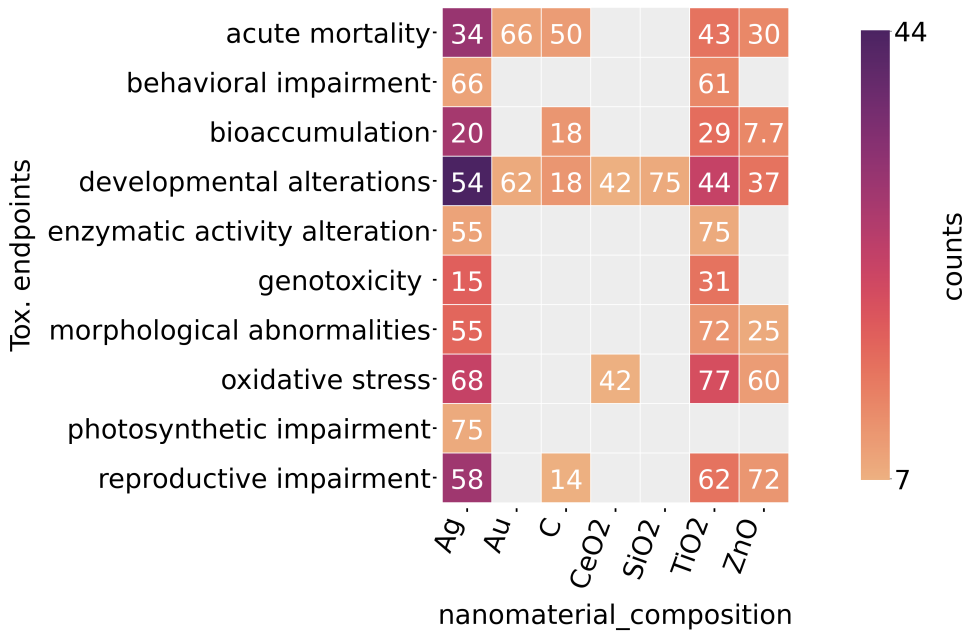

Figure S 13: Heatmap showing the percentage of the LLM positive answers when asked about the possible toxicity of nanomaterials considering varied endpoints. Heatmaps display the nanomaterials composition as a function of the toxicity endpoints for all biological species present in the corpus. The colors indicate the number (counts) of sentences that were analyzed by the LLM while the number inside the boxes indicate the percentage of positive answers from the model (LLM). Percentages were calculated when at least 7 sentences were analyzed for a determined nanomaterial composition and a toxicity endpoint.

2.5 Extracted values - Full tabular data

Table S 7: Numerical parameters extracted for different compositions of nanomaterials.

Composition	*size*	*s_area*	*z_pot*	*log_red*	*MIC*	*MBC*	*BKP*
Ag	27.41<br>(17.0)<br>[22.0]<br>|14.0 : 37.0|	114.53<br>(109.57)<br>[75.44]<br>|35.58 : 148.5|	−21.77<br>(11.6)<br>[−23.4]<br>| − 30.0 : −16.58|	4.66<br>(1.45)<br>[5.0]<br>|3.0 : 6.0|	212.56<br>(1035.09)<br>[15.0]<br>|6.25 : 37.25|	234.1<br>(1015.97)<br>[16.97]<br>|9.46 : 50.0|	74.28<br>(16.67)<br>[77.72]<br>|60.79 : 89.29|
Ag_2O	36.38<br>(25.51)<br>[26.0]<br>|13.0 : 50.0|						
Ag_2S	17.5<br>(10.02)<br>[14.5]<br>|11.5 : 18.5|						
Al	15.62<br>(3.94)<br>[16.0]<br>|12.75 : 18.25|						
Al_2O_3	41.97<br>(30.15)<br>[31.5]<br>|15.0 : 75.0|						
Au	24.33<br>(15.46)<br>[20.0]<br>|12.0 : 35.0|		−7.0<br>(22.35)<br>[−16.8]<br>| − 25.05 : 13.3|	3.19<br>(1.19)<br>[3.5]<br>|2.57 : 4.0|	112.26<br>(201.01)<br>[25.0]<br>|9.14 : 100.0|		58.34<br>(16.89)<br>[57.22]<br>|47.5 : 65.0|
Bi_2O_3	43.0<br>(19.41)<br>[35.0]<br>|29.0 : 55.5|						
C	24.08<br>(69.27)<br>[5.0]<br>|3.0 : 12.0|		−1.64<br>(26.27)<br>[−2.8]<br>| − 27.77 : 20.42|				
CdO	43.17<br>(36.19)<br>[34.5]<br>|21.75 : 44.25|						
CdS	9.33<br>(6.68)<br>[7.0]<br>|5.0 : 12.0|						
CdSe	9.28<br>(10.16)<br>[5.0]<br>|3.38 : 7.75|						
CeO_2	20.48<br>(15.05)<br>[15.5]<br>|9.75 : 28.0|						

Composition	$size$	s_area	z_pot	log_red	MIC	MBC	BKP
Co	40.75<br>(43.75)<br>[21.0]<br>|17.25 : 38.25|						
Co_3O_4	28.36<br>(4.29)<br>[30.0]<br>|25.5 : 30.5|						
$CoFe_2O_4$	25.45<br>(10.97)<br>[25.0]<br>|18.0 : 34.5|						
Cu	37.96<br>(27.24)<br>[30.0]<br>|17.0 : 51.5|		−22.36<br>(15.65)<br>[−23.1]<br>| − 30.0 : −15.4|	5.29<br>(1.73)<br>[5.2]<br>|4.5 : 6.28|	312.24<br>(588.15)<br>[80.0]<br>|32.0 : 200.0|		
Cu_2O	55.83<br>(45.08)<br>[34.5]<br>|28.75 : 82.5|				23.98<br>(18.3)<br>[23.44]<br>|9.3 : 31.44|		
CuO	37.12<br>(25.81)<br>[30.0]<br>|20.0 : 46.75|	230.52<br>(273.1)<br>[48.69]<br>|20.8 : 353.82|	−11.21<br>(20.5)<br>[−20.0]<br>| − 22.55 : −1.23|		193.6<br>(335.7)<br>[32.0]<br>|11.25 : 170.0|		
Fe	43.66<br>(28.99)<br>[37.75]<br>|20.0 : 57.0|						
Fe_2O_3	35.67<br>(20.51)<br>[30.0]<br>|19.0 : 50.0|						
Fe_3O_4	25.24<br>(18.36)<br>[18.5]<br>|11.0 : 33.5|	118.87<br>(127.44)<br>[67.0]<br>|53.06 : 113.6|	−22.23<br>(11.16)<br>[−21.0]<br>| − 30.1 : −14.24|				
Fe_XO_Y	35.55<br>(26.62)<br>[27.0]<br>|13.5 : 53.5|						
Mg	38.25<br>(26.32)<br>[22.0]<br>|19.5 : 64.0|						
MgO	33.54<br>(18.17)<br>[25.0]<br>|20.0 : 50.0|				1191.9<br>(1503.89)<br>[500.0]<br>|321.0 : 1000.0|		
MnO	49.71<br>(22.08)<br>[53.0]<br>|33.5 : 60.0|						

Composition	*size*	*s_area*	*z_pot*	*log_red*	*MIC*	*MBC*	*BKP*
MnO_2	25.86<br>(11.1)<br>[20.0]<br>|17.5 : 33.5|						
MoS_2	27.62<br>(31.9)<br>[15.5]<br>|2.75 : 35.75|						
Ni	49.94<br>(39.3)<br>[29.5]<br>|24.25 : 64.58|						
$NiFe_2O_4$	27.86<br>(15.73)<br>[31.0]<br>|15.5 : 39.0|						
NiO	29.89<br>(20.28)<br>[26.0]<br>|13.0 : 36.0|						
Pd	15.19<br>(14.73)<br>[11.5]<br>|5.0 : 16.5|						
Pt	28.16<br>(26.15)<br>[20.0]<br>|5.0 : 52.0|						
Se	101.08<br>(81.02)<br>[69.0]<br>|37.75 : 150.0|				128.04<br>(150.68)<br>[62.5]<br>|15.62 : 125.0|		
SiO_2	84.17<br>(65.23)<br>[60.0]<br>|35.0 : 125.0|	364.86<br>(135.29)<br>[378.31]<br>|315.67 : 448.66|	−17.7<br>(27.49)<br>[−26.1]<br>| − 40.0 : −5.2|				
SnO_2	26.89<br>(13.72)<br>[25.5]<br>|16.5 : 31.5|						
Ti	47.5<br>(32.76)<br>[31.5]<br>|21.75 : 78.25|						
TiO_2	41.95<br>(31.87)<br>[30.0]<br>|16.67 : 65.0|	187.62<br>(125.87)<br>[153.94]<br>|116.5 : 203.35|	−18.0<br>(8.06)<br>[−19.0]<br>| − 22.87 : −11.56|	3.75<br>(1.37)<br>[3.45]<br>|2.62 : 5.0|	424.47<br>(1276.36)<br>[42.5]<br>|25.0 : 125.0|		
WO_3	36.75<br>(27.91)<br>[32.5]<br>|16.75 : 45.75|						

Composition	$size$	s_area	z_pot	log_red	MIC	MBC	BKP
Zn	38.79<br>(24.93)<br>[30.0]<br>|21.0 : 50.0|	93.91<br>(84.7)<br>[54.26]<br>|30.55 : 150.0|	−7.21<br>(16.68)<br>[−16.35]<br>| − 19.9 : 7.61|				
ZnO	38.83<br>(23.06)<br>[31.0]<br>|20.0 : 50.0|	66.94<br>(66.75)<br>[43.94]<br>|16.89 : 82.43|	−13.31<br>(17.35)<br>[−19.05]<br>| − 23.48 : −9.16|	3.51<br>(1.33)<br>[3.0]<br>|2.3 : 4.5|	266.51<br>(375.98)<br>[79.0]<br>|35.0 : 256.0|	627.09<br>(807.41)<br>[200.0]<br>|53.12 : 1187.5|	63.25<br>(20.94)<br>[62.0]<br>|48.28 : 84.42|
ZnS	17.14<br>(14.42)<br>[10.5]<br>|6.5 : 26.25|						
ZrO_2	68.58<br>(45.73)<br>[75.5]<br>|16.75 : 109.0|						
graphene	80.11<br>(102.6)<br>[27.0]<br>|9.5 : 103.0|						

Table cells contain mean values, standard deviation (STD) in parentheses, median in brackets and interquartile range (IQR) between vertical bars (|).
Mean, STD, median and IQR values were calculated when at least 7 values were extracted.
$size$ stands for nanomaterial size (nm).
s_area stands for the surface area of nanomaterials (m^2 g^{-1}).
z_pot stands for zeta potential (mV).
log_red stands for microbial log reduction (a.u.) .
MIC stands for minimum inhibitory concentration of microorganisms (mg L^{-1}).
MBC stands for minimum bactericidal concentration of microorganisms (%).
BKP stands for biofilm killing percentage of microorganisms (%).

Table S 8: Numerical parameters extracted for different morphologies of nanomaterials.

Morphology	*size*	*z_pot*	*MIC*	*s_area*	*log_red*	*MBC*	*BKP*
dot	5.45<br>(2.94)<br>[5.0]<br>|3.0 : 7.0|	−1.79<br>(26.64)<br>[−2.8]<br>| − 25.78 : 21.28|	240.15<br>(361.86)<br>[63.25]<br>|27.34 : 250.0|				
nanocube	58.44<br>(33.44)<br>[50.0]<br>|50.0 : 80.0|		9.39<br>(11.62)<br>[4.95]<br>|2.36 : 10.0|				
nanofiber	125.68<br>(119.19)<br>[92.5]<br>|20.0 : 180.0|			112.56<br>(124.94)<br>[53.06]<br>|32.05 : 133.24|			
nanoparticle	29.83<br>(19.03)<br>[24.0]<br>|15.0 : 40.0|	−18.74<br>(14.06)<br>[−21.51]<br>| − 27.5 : −14.99|	76.67<br>(127.92)<br>[25.0]<br>|8.0 : 80.0|	114.15<br>(106.14)<br>[78.0]<br>|38.38 : 148.52|	4.05<br>(1.39)<br>[4.0]<br>|3.0 : 5.3|	402.67<br>(1457.93)<br>[32.0]<br>|10.11 : 100.0|	72.26<br>(18.28)<br>[76.34]<br>|54.72 : 90.0|
nanorod	58.29<br>(42.49)<br>[47.0]<br>|24.25 : 80.0|		632.55<br>(1506.59)<br>[38.62]<br>|11.35 : 251.22|		3.88<br>(1.83)<br>[4.0]<br>|2.75 : 4.5|		
nanosheet	60.71<br>(100.29)<br>[20.0]<br>|8.0 : 50.75|			278.45<br>(256.28)<br>[209.36]<br>|53.17 : 419.18|			
nanosphere	68.49<br>(67.86)<br>[50.0]<br>|20.0 : 79.0|		28.56<br>(10.88)<br>[32.0]<br>|20.0 : 38.75|				
nanostructure	37.22<br>(25.95)<br>[29.0]<br>|18.0 : 50.0|		90.36<br>(132.64)<br>[25.0]<br>|7.5 : 93.75|		3.28<br>(1.81)<br>[3.4]<br>|2.22 : 4.5|		
nanotube	58.42<br>(39.17)<br>[55.0]<br>|20.0 : 100.0|			135.99<br>(103.45)<br>[113.5]<br>|49.17 : 215.98|	4.84<br>(2.12)<br>[5.28]<br>|4.15 : 6.28|		
nanowire	79.9<br>(60.89)<br>[75.0]<br>|46.0 : 90.0|						

Table cells contain mean values, standard deviation (STD) in parentheses, median in brackets and interquartile range (IQR) between vertical bars (|).

Mean, STD, median and IQR values were calculated when at least 7 values were extracted.

size stands for nanomaterial size (nm).

s_area stands for the surface area of nanomaterials (m^2 g^{-1}).

z_pot stands for zeta potential (mV).

log_red stands for microbial log reduction (a.u.) .

MIC stands for minimum inhibitory concentration of microorganisms (mg L^{-1}).

MBC stands for minimum bactericidal concentration of microorganisms (%).

BKP stands for biofilm killing percentage of microorganisms (%).

Table S 9: Numerical parameters extracted for different organisms species tested against nanomaterials.

Species	*log_red*	*MIC*	*MBC*	*BKP*
		Ag		
Acinetobacter baumannii		56.96<br>(83.35)<br>[7.8]<br>|2.35 : 64.0|		
Bacillus cereus		42.38<br>(88.26)<br>[8.12]<br>|6.25 : 30.0|		
Bacillus subtilis		94.16<br>(309.1)<br>[25.0]<br>|6.64 : 38.0|		
Candida albicans		60.37<br>(118.21)<br>[22.5]<br>|9.78 : 50.0|		
Enterococcus faecalis		11.36<br>(11.14)<br>[5.19]<br>|4.0 : 15.0|		
Escherichia coli	4.77<br>(1.51)<br>[5.0]<br>|3.0 : 6.0|	33.43<br>(47.58)<br>[15.0]<br>|7.0 : 34.75|	74.63<br>(169.1)<br>[18.53]<br>|10.0 : 44.5|	77.89<br>(14.59)<br>[80.68]<br>|70.09 : 90.0|
Klebsiella pneumoniae		118.35<br>(333.2)<br>[8.0]<br>|5.21 : 47.4|	26.36<br>(25.21)<br>[15.75]<br>|3.95 : 47.25|	
Listeria monocytogenes		139.32<br>(146.78)<br>[100.0]<br>|5.0 : 212.0|		
Pseudomonas aeruginosa		167.59<br>(862.78)<br>[8.0]<br>|3.53 : 16.0|	22.48<br>(20.73)<br>[12.5]<br>|7.96 : 39.0|	76.9<br>(16.49)<br>[76.7]<br>|63.02 : 94.92|
Salmonella typhimurium		93.2<br>(130.41)<br>[31.0]<br>|19.0 : 101.5|		
Staphylococcus aureus	4.21<br>(1.37)<br>[4.0]<br>|3.0 : 5.15|	46.56<br>(67.62)<br>[16.0]<br>|7.72 : 50.0|	113.52<br>(375.03)<br>[24.0]<br>|10.06 : 49.5|	70.23<br>(15.66)<br>[72.7]<br>|54.45 : 82.96|
Staphylococcus epidermidis		10.06<br>(8.65)<br>[8.26]<br>|4.45 : 10.62|		

Species	log_red	MIC	MBC	BKP
**Au**				
Escherichia coli	47.65<br>(55.29)<br>[25.0]<br>|11.2 : 75.0|			
Pseudomonas aeruginosa	212.89<br>(274.9)<br>[75.0]<br>|13.1 : 318.5|			
Staphylococcus aureus	64.61<br>(130.7)<br>[15.3]<br>|6.97 : 43.75|			
**Cu**				
Escherichia coli	174.38<br>(260.79)<br>[41.0]<br>|5.5 : 193.75|			
Staphylococcus aureus	157.0<br>(201.92)<br>[80.0]<br>|24.0 : 170.0|			
**TiO_2**				
Escherichia coli	3.66<br>(1.53)<br>[3.5]<br>|2.5 : 5.1|	32.16<br>(20.08)<br>[35.62]<br>|14.06 : 40.0|		
Staphylococcus aureus		817.51<br>(1960.65)<br>[43.75]<br>|12.96 : 197.5|		
**ZnO**				
Bacillus subtilis		670.27<br>(1710.17)<br>[20.0]<br>|13.0 : 128.0|		
Escherichia coli	4.43<br>(1.46)<br>[4.25]<br>|3.25 : 5.58|	245.87<br>(329.05)<br>[100.0]<br>|27.5 : 252.44|	662.68<br>(913.36)<br>[200.0]<br>|58.0 : 900.0|	58.49<br>(24.44)<br>[51.0]<br>|41.92 : 72.54|
Klebsiella pneumoniae		58.12<br>(65.9)<br>[40.0]<br>|12.88 : 55.5|		
Pseudomonas aeruginosa		628.43<br>(1306.44)<br>[125.0]<br>|40.0 : 375.0|		

Species	log_red	MIC	MBC	BKP
Staphylococcus aureus		349.61<br>(631.68)<br>[65.25]<br>|30.94 : 312.12|	1054.33<br>(1151.58)<br>[200.0]<br>|64.0 : 2000.0|	64.45<br>(23.86)<br>[70.26]<br>|46.5 : 80.7|

Table cells contain mean values, standard deviation (STD) in parentheses, median in brackets and interquartile range (IQR) between vertical bars (|).

Mean, STD, median and IQR values were calculated when at least 7 values were extracted.

log_red stands for microbial log reduction (a.u.) .

MIC stands for minimum inhibitory concentration of microorganisms (mg L^{-1}).

MBC stands for minimum bactericidal concentration of microorganisms (%).

BKP stands for biofilm killing percentage of microorganisms (%).
